# Cell therapy strategies for COVID-19: Current approaches and potential applications

**DOI:** 10.1126/sciadv.abg5995

**Published:** 2021-08-11

**Authors:** Mark M. Zaki, Emal Lesha, Khaled Said, Kiavash Kiaee, Lindsey Robinson-McCarthy, Haydy George, Angy Hanna, Evan Appleton, Songlei Liu, Alex H. M. Ng, Parastoo Khoshakhlagh, George M. Church

**Affiliations:** 1 GC Therapeutics Inc., Cambridge, MA 02139, USA.; 2 Department of Neurosurgery, University of Michigan, 1500 E Medical Center Dr., Ann Arbor, MI 48109, USA.; 3 Department of Neurosurgery, University of Tennessee Health Science Center, Memphis, TN 38163, USA.; 4 Department of Genetics, Harvard Medical School, Boston, MA 02115, USA.; 5 Department of Medicine, Beaumont Hospital, Royal Oak, MI, USA.; 6 Wyss Institute for Biologically Inspired Engineering, Harvard University, Cambridge, MA 02115, USA.

## Abstract

Coronavirus disease 2019 (COVID-19) continues to burden society worldwide. Despite most patients having a mild course, severe presentations have limited treatment options. COVID-19 manifestations extend beyond the lungs and may affect the cardiovascular, nervous, and other organ systems. Current treatments are nonspecific and do not address potential long-term consequences such as pulmonary fibrosis, demyelination, and ischemic organ damage. Cell therapies offer great potential in treating severe COVID-19 presentations due to their customizability and regenerative function. This review summarizes COVID-19 pathogenesis, respective areas where cell therapies have potential, and the ongoing 89 cell therapy trials in COVID-19 as of 1 January 2021.

## Teaser

Cell therapy has strong promise for treating COVID-19 survivors due to customizability, targetability, and regenerative function.

## INTRODUCTION

Coronavirus disease 2019 (COVID-19) continues to strain patients, providers, and health care systems worldwide. Since its discovery, the disease has contributed to approximately 200 million infections and 4 million deaths worldwide. The scientific community has focused vast resources on understanding the virus causing COVID-19, named severe acute respiratory syndrome coronavirus 2 (SARS-CoV-2), and the pathologies associated with the infection. Enormous effort has been placed to shed light on the mechanisms of viral entry and infection, the interaction between the virus and the host’s immune system, and the mechanisms of injury underlying the common manifestations of the disease.

SARS-CoV-2 initially emerged as a pathogen causing mainly viral pneumonias; however, experience in the proceeding months showed that the disease manifests throughout the body, leading to pathologies of the immune, renal, cardiac, and nervous systems, among others. While most patients have a mild course, over 15% develop severe and critical disease (*1*), leading to a substantial number of patients requiring prolonged hospitalization with intensive care services and potentially facing subsequent chronic manifestations related to pathological injuries from the disease process. In addition, mortality can be as high as 61.5% in critically ill patients with the disease (*2*).

As we begin to appreciate the subacute and chronic sequela of COVID-19, it is crucial to focus research efforts on finding therapies that not only dampen the acute damage but also can do so in a targeted manner while restoring physiological function and addressing the long-term sequela of the disease. Cell therapies have the potential to regenerate damaged tissue and tackle the immune system and, hence, are a treatment option with great promise. Here, we provide an overview of the COVID-19 pathogenesis in various organ systems, the overall advantages of cell therapies, potential cell targets and strategies within each organ system, and a summary of current cell therapy studies and trials for COVID-19 as of 1 January 2021.

## VIRAL ENTRY AND TROPISM

SARS-CoV-2 first interacts with cells via binding of the viral spike protein to angiotensin-converting enzyme 2 (ACE2) on the cell surface (*3*, *4*). After binding to ACE2, the spike protein is processed by the host transmembrane protease serine 2 (TMPRSS2), priming it for membrane fusion. This is considered to be the primary route of infection in vivo. Alternatively, the virus can be taken up into the cell via endocytosis and the spike protein processed by the endosomal proteases cathepsins B and L (*3*). After fusion with the cell membrane and release into the cytoplasm, the RNA replication machinery encoded in the first open reading frame of the viral genome is translated, followed by RNA replication and viral protein translation. SARS-CoV-2 co-opts and alters numerous cellular proteins and pathways, many of which are yet to be elucidated (*5*). It has been indicated that neuropilin 1 (NRP1) has a role in potentiating SARS-CoV-2 entry through the ACE2 pathway (*6*, *7*). Studies from other coronaviruses provided evidence for CD147 and the 78-kDa glucose-regulated protein (GRP78) as putative alternative receptors, but more investigations on how the collective tissue distribution of these factors correlate with viral tropism and disease symptoms are under active investigation (*8*, *9*).

Cellular tropism of SARS-CoV-2 is considered to be largely dictated by the distribution of ACE2. Bulk transcriptomic studies found ACE2 primarily expressed in the lungs, intestinal tract, kidneys, gallbladder, and heart; lower levels of expression were observed in the brain, thyroid, adipose tissue, epididymis, ductus deferens, breast, pancreas, rectum, ovary, esophagus, liver, seminal vesicle, salivary gland, placenta, vagina, lung, appendix, and skeletal muscle (*10*–*12*). In the respiratory tract, ACE2 is most highly expressed in nasal epithelial cells, where SARS-CoV-2 is thought to initially infect followed by propagation into the distal alveoli (*13*). Many organs that express higher levels of ACE2 are not major sites of viral replication, indicating that expression of other host factors, including TMPRSS2, NRP1, and host restriction factors likely contributes to viral tropism (*12*).

## ADVANTAGES AND POTENTIAL FOR CELL THERAPIES

Although most patients infected with SARS-CoV-2 present with mild symptoms (*14*), a considerable part of the population, including elderly patients and those with underlying comorbidities, have an increased risk of more severe outcomes, including death (*15*). Current treatment options for severely ill patients, aimed at reducing inflammation during the acute phase of the infection, have their limitations. Medications may be nonspecific for SARS-CoV-2 targets or are repurposed without a clear mechanism of benefit, while others such as remdesivir and tocilizumab may not be readily accessible because of federal allocations or cost barriers (*16*). In addition, these treatments have not focused on long-term sequela of the disease such as regeneration of damaged tissue structure and function. Cell therapies may thus be a promising class of therapies that could overcome these challenges through their customizability, targetability, scalable manufacturing, and restoration of function.

Cellular therapies have shown success in treating conditions that have otherwise been challenging to manage with mainstream treatment modalities, including, but not limited to, oncologic, neurodegenerative, and immunologic disorders. Cell therapy approaches including, but not limited to, mesenchymal stromal cells (MSCs), induced pluripotent stem cells (iPSCs), and T cells have been widely studied, and their efficacy has led to several U.S. Food and Drug Administration (FDA) approvals of cell therapies including, most famously, axicabtagene ciloleucel (Yescarta) and tisagenlecleucel (Kymriah) (*17*–*20*). Extensive safety and efficacy data from cell therapies trials in various indications suggest that cell therapies could play a role in treating patients with COVID-19 as well.

Two potential concerns with cell therapies are immune rejection and tumorigenicity. Immune rejection concerns for allogeneic cell therapy have been discussed in the literature, especially as new cell therapies emerge. MSCs, for example, are considered to be immune suppressive and immune evasive, yet, the standard of treatment using allogeneic MSCs is the addition of immunosuppressive regimens alongside the cell therapy (*21*, *22*). While immunosuppressive therapy may be used to protect the graft, it may not always prevent graft rejection and can come with its own adverse effects. Genome engineering can help address the immune system by tackling both the innate and adaptive immune systems. Potential strategies include knocking out genes responsible for immune system activation, such as major histocompatibility complex I and II (*23*, *24*). These modifications could address both the acute and chronic rejection phases, making the cell grafts more resistant to the host immune system.

Tumorigenicity is an important consideration with cell therapies. The risk of tumorigenicity seems to be greater with MSCs, iPSCs, and human embryonic stem cells (hESCs), and it can present in the form of teratoma or as a true tumor (*25*–*27*). This risk can be reduced by increasing the efficiency of differentiation to the target cell type thereby reducing residual pluripotent cells, such as by transcription factor–mediated cell programming or by incorporating suicide genes into cell grafts that can be activated in the rare chance a graft becomes malignant (*28*–*30*). Several suicide mechanisms have been described in the literature, including a recent study by Itakura *et al.* (*31*) in which iCaspase9 was inserted as a fail-safe system in iPSC cell lines. If these cell lines become cancerous once transplanted in mice, induction of the iCaspase9 with a small molecule showed the formed tumors to rapidly reduce in size (*31*). These approaches increase the safety profile of cell therapies for clinical applications in patients with COVID-19 and beyond.

## COVID-19 PATHOGENESIS AND CELL THERAPY POTENTIAL BY ORGAN SYSTEM

**Fig. 1:**
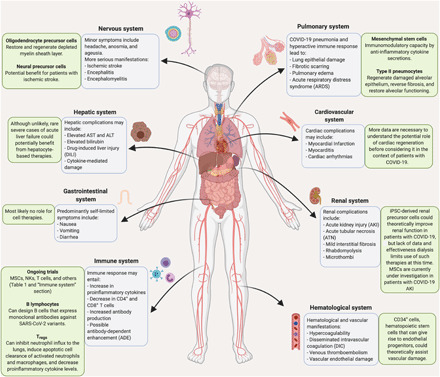
Summary of COVID-19 pathogenesis and respective potential for cell therapies by organ system. Blue text boxes describe specific pathogenesis for each organ system. Green text boxes describe potential and ongoing cell therapy applications for each organ system. ALT, alanine aminotransferase; AST, aspartate aminotransferase.

**Fig. 2:**
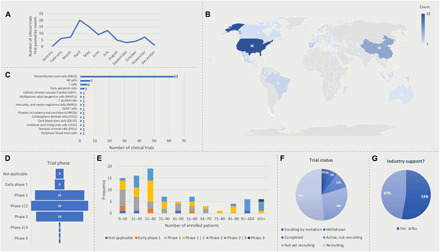
Descriptive summary of the 89 clinical trials using cell therapies for COVID-19. (**A**) Number of COVID-19 targeting cell therapy clinical trials started in each month of the year 2020. (**B**) World map showing global distribution of the registered cell therapy clinical trials and their numbers per country. (**C**) Different cell types used in the cell therapy–based clinical trials and their respective count. (**D**) Stages of the 89 cell therapy clinical trials registered as of 1 January 2021. (**E**) Distribution of patient enrollment numbers across the 89 clinical trials. (**F**) Breakdown of the 89 cell therapy clinical trial statuses. (**G**) The percentages of cell therapies sponsored and supported by the industry sector.

A clear understanding of COVID-19 pathogenesis is necessary to appreciate the potential benefit of cell therapies. Cell therapies provide paramount benefit as potential targeted treatment strategies to address localized damage inflicted by the disease and restore physiological functions (Fig. 1). In 2020, March and April recorded a large initial surge in global COVID-19 cases and deaths, as presented by the World Health Organization. There was a concurrent increase in the numbers of cell therapy–based clinical trials initiated during those 2 months (Fig. 2A). As of 1 January 2021, there are 89 cell therapy–based clinical trials registered on clinicaltrials.gov (Table 1) targeting COVID-19 pathology. Most of the clinical trials are held in the United States and China, 36% and 16%, respectively, with the rest of the clinical trials spread across the globe (Fig. 1B). MSCs constitute the majority cell type used in the cell therapy clinical trials, around 71%, with the rest using cell types such as natural killer (NK) cells, T cells, early apoptotic cells, and others (Fig. 1C). About 88% of the clinical trials are in phases 1 and 2, with one trial in phase 2/3 and one in phase 3 (Fig. 2D). The enrollment in each clinical trial was most frequently 21 to 30 patients but ranged up to 400 depending on the phase of the trial (Fig. 2E). In addition, the variability of patient enrollment numbers could be due to the varying statuses of each clinical trial (Fig. 2F). It is also worth noting that over half of the cell therapy–based clinical trials are sponsored and supported by the industry sector (Fig. 2G), which indicates the pivotal role for industry in accelerating the necessary research to combat COVID-19.

**Table 1:** Cell therapy–based clinical trials for COVID-19. Search approach: performed 1 January 2021; Clinicaltrials.gov: advanced search; Condition - –OVID; Study Type -Interventi–nal; Intervention/treatment - Cell; of 157 studies, exclude– non–COVID-19 patients (*n* = 12) and non–cell therapy trials (*n* = 56); leaving 89 available studies. NCT, national clinical trial.

**Cell type**	**Title**	**Status**	**Phase**	**Country**	**NCT number**
**Mesenchymal** **stem cells**					
	Clinical trial of allogeneic mesenchymal cells from umbilical cord tissue in patients with COVID-19	Recruiting	Phase 2	Spain	NCT04366271
	Autologous adipose- derived stem cells (AdMSCs) for COVID-19	Not yet recruiting	Phase 2	United States	NCT04428801
	Treatment of severe COVID-19 pneumonia with allogeneic mesenchymal stromal cells (COVID_MSV)	Recruiting	Phase 2	Spain	NCT04361942
	Mesenchymal stem cell infusion for COVID-19 infection	Recruiting	Phase 2	Pakistan	NCT04444271
	Mesenchymal stem cell for acute respiratory distress syndrome due for COVID-19	Recruiting	Phase 2	Mexico	NCT04416139
	Safety and feasibility of allogenic MSC in the treatment of COVID-19	Not yet recruiting	Phase 1	Brazil	NCT04467047
	BAttLe against COVID-19 using mesenchYmal stromal cells	Not yet recruiting	Phase 2	Spain	NCT04348461
	Mesenchymal stromal cells for the treatment of SARS-CoV-2 induced acute respiratory failure (COVID-19 disease)	Not yet recruiting	Early phase 1	United States	NCT04345601
	Cellular immuno- therapy for COVID-19 acute respiratory distress syndrome - Vanguard	Recruiting	Phase 1	Canada	NCT04400032
	Cord blood-derived mesenchymal stem cells for the treatment of COVID-19 related acute respiratory distress syndrome	Recruiting	Phase 1	United States	NCT04565665
	Safety and efficacy study of allogeneic human dental pulp mesenchymal stem cells to treat severe COVID-19 patients	Recruiting	Phase 1|phase 2	China	NCT04336254
	Study of intravenous administration of allogeneic adipose stem cells for COVID-19	Not yet recruiting	Phase 1	United States	NCT04486001
	Safety and efficacy of mesenchymal stem cells in the management of severe COVID-19 pneumonia	Not yet recruiting	Phase 2	Colombia	NCT04429763
	NestaCell mesenchymal stem cell to treat patients with severe COVID-19 pneumonia	Not yet recruiting	Phase 2	Brazil	NCT04315987
	Use of mesenchymal stem cells in acute respiratory distress syndrome caused by COVID-19	Active, not recruiting	Early phase 1	Mexico	NCT04456361
	Efficacy of infusions of MSC from Wharton jelly in the SARS-Cov-2 (COVID-19) related acute respiratory distress syndrome	Not yet recruiting	Phase 2	France	NCT04625738
	hCT-MSCs for COVID19 ARDS	Recruiting	Phase 1|phase 2	United States	NCT04399889
	Clinical trial to assess the efficacy of MSC in patients with ARDS due to COVID-19	Recruiting	Phase 2	Spain	NCT04615429
	Treatment of Covid-19 associated pneumonia with allogenic pooled olfactory mucosa- derived mesenchymal stem cells	Enrolling by invitation	Phase 1|phase 2	Belarus	NCT04382547
	Clinical trial to assess the safety and efficacy of intravenous administration of allogeneic adult mesenchymal stem cells of expanded adipose tissue in patients with severe pneumonia due to COVID-19	Active, not recruiting	Phase 1|phase 2	Spain	NCT04366323
	Regenerative medicine for COVID-19 and flu-elicited ARDS using longeveron mesenchymal stem cells (LMSCs) (RECOVER)	Recruiting	Phase 1	United States	NCT04629105
	An exploratory study of ADR-001 in patients with severe pneumonia caused by SARS-CoV-2 infection	Not yet recruiting	Phase 1	Japan	NCT04522986
	Novel coronavirus induced severe pneumonia treated by dental pulp mesenchymal stem cells	Not yet recruiting	Early phase 1	China	NCT04302519
	Multiple dosing of mesenchymal stromal cells in patients with ARDS (COVID-19)	Recruiting	Phase 2	United States	NCT04466098
	MSC-based therapy in COVID-19-associated acute respiratory distress syndrome	Recruiting	Phase 1	Brazil	NCT04525378
	Adipose mesenchymal cells for abatement of SARS-CoV-2 respiratory compromise in COVID-19 disease	Not yet recruiting	Phase 1	United States	NCT04352803
	Safety and efficacy of intravenous Wharton’s jelly derived mesenchymal stem cells in acute respiratory distress syndrome due to COVID 19	Not yet recruiting	Phase 1|phase 2	Colombia	NCT04390152
	Efficacy of intravenous infusions of stem cells in the treatment of COVID-19 patients	Recruiting	Phase 2	Pakistan	NCT04437823
	Treatment with human umbilical cord- derived mesenchymal stem cells for severe corona virus disease 2019 (COVID-19)	Completed	Phase 2	China	NCT04288102
	Therapeutic study to evaluate the safety and efficacy of DW-MSC in COVID-19 patients	Active, not recruiting	Phase 1	Indonesia	NCT04535856
	Administration of allogenic UC-MSCs as adjuvant therapy for critically-ill COVID-19 patients	Recruiting	Phase 1	Indonesia	NCT04457609
	Clinical research of human mesenchymal stem cells in the treatment of COVID-19 pneumonia	Recruiting	Phase 1|phase 2	China	NCT04339660
	Treatment of COVID-19 patients using Wharton’s jelly-mesenchymal stem cells	Recruiting	Phase 1	Jordan	NCT04313322
	Study of human umbilical cord mesenchymal stem cells in the treatment of severe COVID-19	Not yet recruiting	Not applicable	China	NCT04273646
	Mesenchymal stromal cell therapy for severe Covid-19 infection	Recruiting	Phase 1|phase 2	Belgium	NCT04445454
	Bone marrow-derived mesenchymal stem cell treatment for severe patients with coronavirus disease 2019 (COVID-19)	Not yet recruiting	Phase 1|phase 2	China	NCT04346368
	A study of cell therapy in COVID-19 subjects with acute kidney injury who are receiving renal replacement therapy	Recruiting	Phase 1|phase 2	United States	NCT04445220
	Mesenchymal stem cells in patients diagnosed with COVID-19	Recruiting	Phase 1	Mexico	NCT04611256
	Use of UC-MSCs for COVID-19 patients	Completed	Phase 1|phase 2	United States	NCT04355728
	Mesenchymal stem cell treatment for pneumonia patients infected with COVID-19	Recruiting	Phase 1	China	NCT04252118
	Study to evaluate the efficacy and safety of AstroStem-V in treatment of COVID-19 pneumonia	Not yet recruiting	Phase 1|phase 2	South Korea	NCT04527224
	A randomized, double-blind, placebo-controlled clinical trial to determine the safety and efficacy of Hope Biosciences allogeneic mesenchymal stem cell therapy (HB-adMSCs) to provide protection against COVID-19	Enrolling by invitation	Phase 2	United States	NCT04348435
	A clinical trial to determine the safety and efficacy of Hope Biosciences autologous mesenchymal stem cell therapy (HB-adMSCs) to provide protection against COVID-19	Active, not recruiting	Phase 2	United States	NCT04349631
	ASC therapy for patients with severe respiratory COVID-19	Withdrawn	Phase 1|phase 2	Denmark	NCT04341610
	Safety and effectiveness of mesenchymal stem cells in the treatment of pneumonia of coronavirus disease 2019	Active, not recruiting	Early phase 1	China	NCT04371601
	Cell therapy using umbilical cord- derived mesenchymal stromal cells in SARS-CoV-2-related ARDS	Active, not recruiting	Phase 1|phase 2	France	NCT04333368
	Clinical use of stem cells for the treatment of Covid-19	Recruiting	Phase 1|phase 2	Turkey	NCT04392778
	Mesenchymal stem cells for the treatment of COVID-19	Completed	Phase 1	United States	NCT04573270
	Treatment of coronavirus COVID-19 pneumonia (pathogen SARS-CoV-2) with cryopreserved allogeneic P_MMSCs and UC-MMSCs	Recruiting	Phase 1|phase 2	Ukraine	NCT04461925
	Efficacy and safety Study of allogeneic HB-adMSCs for the treatment of COVID-19	Active, not recruiting	Phase 2	United States	NCT04362189
	Use of hUC-MSC product (BX-U001) for the treatment of COVID-19 With ARDS	Not yet recruiting	Phase 1|phase 2	United States	NCT04452097
	Mesenchymal stem cells (MSCs) in inflammation- resolution programs of coronavirus disease 2019 (COVID-19) induced acute respiratory distress syndrome (ARDS)	Not yet recruiting	Phase 2	Germany	NCT04377334
	Umbilical cord tissue (UC) derived mesenchymal stem cells (MSCs) versus placebo to treat acute pulmonary inflammation due to COVID-19	Not yet recruiting	Phase 1	United States	NCT04490486
	Repair of acute respiratory distress syndrome by stromal cell administration (REALIST) (COVID-19)	Recruiting	Phase 1|phase 2	United Kingdom	NCT03042143
	The MEseNchymal coviD-19 trial: A pilot study to investigate early efficacy of MSCs in adults with COVID-19	Recruiting	Phase 1|phase 2	Australia	NCT04537351
	Efficacy and safety evaluation of mesenchymal stem cells for the treatment of patients with respiratory distress due to COVID-19	Recruiting	Phase 1|phase 2	Spain	NCT04390139
	Study of the safety of therapeutic Tx with immunomodulatory MSC in adults with COVID-19 infection requiring mechanical ventilation	Recruiting	Phase 1	United States	NCT04397796
	Open-label multicenter study to evaluate the efficacy of PLX-PAD for the treatment of COVID-19	Recruiting	Phase 2	Germany	NCT04614025
	MSCs in COVID-19 ARDS	Active, not recruiting	Phase 3	United States	NCT04371393
	Therapy for pneumonia patients iInfected by 2019 novel coronavirus	Withdrawn	Not applicable	China	NCT04293692
	Umbilical cord(UC)- derived mesenchymal stem cells (MSCs) treatment for the 2019-novel coronavirus (nCOV) pneumonia	Recruiting	Phase 2	China	NCT04269525
	ACT-20 in patients with severe COVID-19 pneumonia	Not yet recruiting	Phase 1|phase 2	United States	NCT04398303
	Double-blind, multicenter, study to evaluate the efficacy of PLX PAD for the treatment of COVID-19	Recruiting	Phase 2	United States	NCT04389450
**Natural killer (NK) cells**					
	Fase I clinical trial on NK cells for COVID-19	Not yet recruiting	Phase 1	Brazil	NCT04634370
	A phase I/II study of universal off-the- shelf NKG2D-ACE2 CAR-NK Cells for therapy of COVID-19	Recruiting	Phase 1|phase 2	China	NCT04324996
	Phase I / II clinical study of immunotherapy based on adoptive cell transfer as a therapeutic alternative for patients with COVID-19 in Colombia	Not yet recruiting	Phase 1|phase 2	Colombia	NCT04344548
	NK cells treatment for COVID-19	Recruiting	Phase 1	China	NCT04280224
	Natural killer cell (CYNK-001) infusions in adults with COVID-19	Recruiting	Phase 1|phase 2	United States	NCT04365101
	Study of FT516 for the treatment of COVID-19 in hospitalized patients with hypoxia	Recruiting	Phase 1	United States	NCT04363346
	An experiment to evaluate the safety of agenT-797 in COVID-19 patients with severe difficulty breathing	Recruiting	Phase 1	United States	NCT04582201
**T cells**					
	Part two of novel adoptive cellular therapy with SARS-CoV-2 specific T cells in patients with severe COVID-19	Recruiting	Phase 1|phase 2	Singapore	NCT04457726
	RAPA-501-Allo off-the-shelf therapy of COVID-19	Recruiting	Phase 1|phase 2	United States	NCT04482699
	REgulatory T cell infuSion fOr lung injury due to COVID-19 PnEumonia	Recruiting	Phase 1	United States	NCT04468971
	Anti-SARS Cov-2 T cell infusions for COVID 19	Recruiting	Phase 1	United States	NCT04401410
	Viral specific T-cells for treatment of COVID-19	Not yet recruiting	Phase 2	United States	NCT04406064
**Early apoptotic cells**					
	Study evaluating safety, tolerability and efficacy of Allocetra-OTS in patients with COVID-19	Not yet recruiting	Phase 1	Israel	NCT04659304
	Allocetra-OTS in COVID-19	Active, not recruiting	Phase 1	Israel	NCT04513470
	Allocetra-OTS in COVID-19, phase II	Recruiting	Phase 2	Israel	NCT04590053
**Other**					
Cardiosphere-derived cells	Intravenous infusion of CAP-1002 in patients with COVID-19	Recruiting	Phase 2	United States	NCT04623671
CD34^+^ cells	CLBS119 for repair of COVID-19 induced pulmonary damage	Withdrawn	Phase 1	United States	NCT04522817
Cellular stromal vascular fraction (cSVF)	Use of cSVF Via IV deployment for residual lung damage after symptomatic COVID-19 infection	Recruiting	Early phase 1	United States	NCT04326036
Cord blood stem cells	Stem cell educator therapy treat the viral inflammation in COVID-19	Not yet recruiting	Phase 2	China	NCT04299152
Decidual stromal cells	Study of decidual stromal cells to treat COVID-19 respiratory failure	Not yet recruiting	Not applicable	Canada	NCT04451291
Immunity- and matrix-regulatory cells	Safety and efficacy of CAStem for severe COVID-19 associated with/without ARDS	Recruiting	Phase 1|phase 2	China	NCT04331613
Multipotent adult progenitor cells	MultiStem administration for COVID-19 induced ARDS (MACoVIA)	Recruiting	Phase 2|phase 3	United States	NCT04367077
Peripheral blood stem cells	Study evaluating the safety and efficacy of autologous non-hematopoietic peripheral blood stem cells in COVID-19	Completed	Phase 1|phase 2	United Arab Emirates	NCT04473170
Platelet-rich plasma and cord blood	Using PRP and cord blood in treatment of Covid -19	Recruiting	Not applicable	Egypt	NCT04393415
T and NK cells	Safety infusion of NatuRal KillEr celLs or MEmory T cells as adoptive therapy in COVID-19 pnEumonia or lymphopenia	Recruiting	Phase 1|phase 2	Spain	NCT04578210
Umbilical cord lining stem cells (ULSCs)	Umbilical cord lining stem cells (ULSC) in patients with COVID-19 ARDS	Recruiting	Phase 1|phase 2	United States	NCT04494386

## PULMONARY SYSTEM

Pulmonary symptoms are the mainstay of COVID-19 and may include dry cough, dyspnea, pneumonia, and acute respiratory distress syndrome (ARDS) (*32*). Bilateral pulmonary infiltrates and ground-glass opacities are seen radiographically in over 70% of hospitalized patients (*14*). Furthermore, ARDS has shown to be present in over 90% of deceased patients (*33*). ARDS and the associated alveolar damage are thought to be primarily due to immune-related response (*3*, *34*). Other pulmonary complications may include secondary pulmonary hypertension, hypercoagulability-related pulmonary emboli, and long-lasting fibrosis in patients who do recover from the acute infection (*35*, *36*).

Some preclinical data suggest that patients with COVID-19 may benefit from cell therapies, particularly using MSCs in models of viral and inflammatory lung damage (*37*). For instance, MSCs were found to reduce the impairment of alveolar fluid clearance caused by influenza A H5N1 infection in vitro and mitigate lung injury in vivo (*38*). Another study showed that MSC treatment reduces influenza H9N2–induced acute lung injury in mice and reduces pulmonary inflammation (*39*). In another study, MSCs were shown to promote macrophages to become anti-inflammatory and take on a phagocytic phenotype through extracellular vesicles, thereby ameliorating lung injury in mice (*40*).

Several studies have described promising treatment of pneumonia and ARDS in critically ill patients with COVID-19 using cell therapies. In China, Liang *et al.* (*41*) reported treatment of one patient with severe COVID-19 unresponsive to steroid medications, after three successive injections of 5 × 10^7^ human umbilical cord MSCs at days 1, 4, and 7 of treatment initiation. The patient’s pulmonary lesions had begun to resolve by day 7 after the first MSC injection. Tang *et al.* (*42*) reported treatment with allogeneic menstrual blood–derived MSCs of two patients with COVID-19 with ARDS. Treatment involved three successive injections of 1 × 10^6^ MSCs/kg of body weight at days 1, 2, and 4 of treatment initiation. Both patients were discharged from the hospital. Leng *et al.* (*43*) reported a pilot study where they transplanted a single dose of 1 × 10^6^ MSCs/kg of body weight in seven patients with mild, severe, and critical COVID-19, with three patients on the placebo arm. Results from the study showed overall safety of the treatment, with two severe patients recovering and being discharged within 10 days of treatment. In Spain, Sanchez-Guijo *et al.* (*44*) treated 10 patients under mechanical invasive intubation with either one, two, or three doses of 1 × 10^6^ adipose-derived MSCs/kg of body weight. Seven of the 13 patients were extubated approximately 7 days after initiation of treatment. Furthermore, the authors observed that patients who received cell therapy earlier in their disease course had better outcomes. These open label–uncontrolled administrations are important as they demonstrate apparent safety with no obvious adverse events.

Various MSC-based strategies are assessing treatment of patients with COVID-19 with pulmonary symptoms, especially pneumonia and ARDS. One phase 1/2a randomized double-blind trial (NCT04355728) assessed administration of two infusions of 1 × 10^7^ umbilical cord–derived MSCs for COVID-19 ARDS, showing improved 28-day survival following therapy (91% in treatment group, *n* = 12 versus 42% in control, *n* = 12) (*45*). Another phase 3 study comparing administration of two injections of 2 × 10^6^ MSCs/kg of body weight and standard of care compared to placebo injection and standard of care in patients with COVID-19 with moderate to severe ARDS failed to meet the primary end point of 43% reduction in mortality in an interim analysis (NCT04371393). Thus, further investigation is necessary to determine whether MSC-based therapy could improve COVID-related lung injury.

COVID-19–related lung fibrosis has been characterized by fibroblast proliferation, airspace obliteration, and microhoneycombing, which is thought to persist in patients who survive the acute infection (*46*). This pattern of fibrotic change may be similar to that of idiopathic pulmonary fibrosis (IPF) (*36*), and prior cell therapy studies in IPF may shed light on potential avenues for cell therapy applications in patients with COVID-19. IPF is a progressive disease of unknown etiology that leads to fibrosis of the lungs and is the primary cause of more than half of all lung transplants worldwide (*47*). Cell therapies using type II pneumocytes (PTIIs), which are progenitors of the lung alveolar epithelium, have shown efficacy in preclinical animal models of IPF by regenerating lung epithelium, releasing surfactant, and reversing pulmonary fibrosis (*48*, *49*). A phase 1 clinical study also showed that targeted intratracheal delivery of PTIIs showed safety and clinical stability at 12-month follow-up of 16 patients with moderate to severe IPF (*50*). In addition to PTIIs, MSCs have also been used in IPF. A recent randomized trial of patients with IPF treated with two doses of 2 × 10^8^ allogenic bone marrow MSCs every 3 months for 1 year showed safety and improved respiratory function when compared to control participants (*51*). This suggests that even patients with COVID-19 with residual chronic fibrosis may benefit from cell-based therapies in the future, although further data are necessary to support this conclusion. Ultimately, cell therapies that can reverse fibrotic changes or supplement normal pneumocyte function could address potential chronic pulmonary effects from COVID-19.

## IMMUNE SYSTEM

The host’s immune response toward SARS-CoV-2 has been studied carefully since the outbreak, with many potential mechanisms of interaction being elucidated on the basis of similarities of the virus to SARS-CoV. Most patients with COVID-19 mount antibody responses to SARS-CoV-2, which vary in magnitude and potency (*52*). Neutralizing antibodies appear to target the receptor binding domain of the spike proteins (*52*, *53*). Patients with high immunoglobulin M (IgM) and immunoglobulin G (IgG) titers have a worse prognosis (*54*), which could be correlated with high viral load but could also indicate a harmful robust immune response through antibody-dependent enhancement (ADE). ADE is a phenomenon that has been observed in several viruses, including SARS-CoV, where viral-specific antibodies promote viral entry into immune cells expressing Fc receptors (*55*), such as monocytes, macrophages, and B cells, leading to enhanced amplification of the virus. Implications of ADE in COVID-19 have been discussed in greater detail by Eroshenko *et al.* (*56*). With regard to T cells, several studies have compared leukocyte profiles between patients with mild and severe manifestations of the disease and showed decreased T cell count in both CD4^+^ and CD8^+^ populations, more commonly in intensive care unit (ICU) patients but highly prevalent in non-ICU patients as well (*57*). Lower levels of CD4^+^ T helper cells and CD8^+^ cytotoxic T cells likely hinder the ability of the immune system to neutralize and kill viral-infected cells.

In addition, a marked increase of proinflammatory cytokines such as interleukin-1 (IL-1), IL-6, tumor necrosis factor–α (TNF-α), and interferon-γ (IFN-γ) has been observed in patients with severe COVID-19 (*57*, *58*). In these cases, SARS-CoV-2 immune evasion leads to a robust viral replication and a delayed and dysregulated IFN-γ response, resulting in recruitment and accumulation of inflammatory macrophages and neutrophils (*58*, *59*). Further IFN-γ activation by these cells leads to additional cytokine and chemokine signals [IFN-γ, TNF-α, C-C motif chemokine ligand (CCL)2, CCL7, and CCL12] that enhance infiltration and activation of monocytes and neutrophils, further exacerbating the inflammatory response and inducing high cytokine levels, a phenomenon referred to as cytokine storm, which has been linked to more severe manifestations of COVID-19 (*60*).

Several immune-based cell strategies can be proposed for targeting different pathologies of COVID-19. Several NK cell therapies for COVID-19 are under investigation (Table 1). NK cells are activated and recruited at the site of infection in response to IL-12 and IL-18 signals. They control viral replication using perforin and granzyme granules and induce Fas ligand– or TNF-a–related apoptosis-inducing ligand–mediated apoptosis in infected cells (*61*). Cell therapies involving NK cells and chimeric antigen receptor (CAR) NK cells have shown clinical safety and efficacy in numerous oncological indications (*62*), and they may have a role in treating various infectious pathologies as well (*63*). As NK cells recognize viral infected cells by identifying up-regulated stress markers and down-regulated inhibitory ligands, exogenous administration of NK cell–based therapies could thus assist in identifying SARS-CoV-2–infected cells and promote viral clearance (*64*). A phase 1 study is assessing the efficacy and safety of CYNK-001 cells, which are allogeneic, off-the-shelf, and cryopreserved NK cells derived from CD34^+^ human placental stem cells, in 14 adult patients with mild to moderate COVID-19 (NCT04365101). In another phase 1 study, FT516 cells, which are allogeneic, off-the-shelf, and cryopreserved NK cells derived from iPSCs, are being tested for efficacy and safety in 12 adult patients with COVID-19 who are hospitalized and fulfill requirements for hypoxia (NCT04363346). With regard to CAR NK cells, a phase 1/2 study in China is using off-the-shelf NKG2D-ACE2 CAR NK cells to target viral infected cells while also secreting IL-15 as a superagonist and granulocyte-macrophage colony-stimulating factor neutralizing single-chain variable fragment to reduce the likelihood of cytokine release syndrome (NCT04324996). Intravenous infusion of 1 × 10^8^ cells/kg of body weight will be administered weekly in patients with COVID-19, and the study is currently recruiting patients.

Given that immune system overactivation is a significant factor in pathologies of COVID-19, another potential strategy could involve CD4^+^CD25^+^Foxp3^+^ regulatory T cells (T_regs_). T_regs_ function by secreting anti-inflammatory cytokines IL-10 and transforming growth factor–β (TGF-β) as well as by contact-dependent signaling, and have been shown to inhibit the influx of neutrophils to the lung, induce apoptotic cell clearance of activated neutrophils and macrophages, and decrease proinflammatory cytokine levels (*65*, *66*). Moreover, they can inhibit excessive innate immune responses via induction of secondary immunosuppressive neutrophils that generate anti-inflammatory cytokines and via enzymes indoleamine 2,3-dioxygenase and heme oxygenase-1, which further inhibit cellular proliferation (*66*). The safety and feasibility of T_regs_ has been clinically evaluated over the past decade, showing tolerability and clinical improvement especially in the setting of solid-organ transplantation and autoimmune disease (*67*). Hence, the immunosuppressive role of T_regs_ may be beneficial in quelling the cytokine storm in patients with COVID-19. Potential strategies may include using polyclonal expanded T_regs_ versus engineered antigen-specific T_reg_ approaches. Polyclonal T_regs_ offer a more generalized immunosuppressive strategy, which may be similar to current immunosuppressive medications. Polyclonal T_regs_ have been clinically evaluated with promising results in type 1 diabetes and other autoimmune diseases (*68*), but they have not been clinically tested in immune overactivation in viral infections. A concern with this therapy would be the exacerbation of acute infection by excessive quelling of the host immune response to SARS-CoV-2. Engineered antigen-specific T_regs_ could help localize immunosuppressive effects (*65*), but this could also facilitate enhanced viral replication. Overall, T_reg_ therapies could aid in suppressing the overactive immune system in patients with COVID-19 (*69*), but generalizing early safety data from clinical trials of autoimmune and transplant patients toward patients with COVID-19 would need careful evaluation. Two phase 1 clinical trials, which are not yet recruiting, are aiming to test the efficacy and safety of allogeneic, off-the-shelf, and cryopreserved T_reg_ cell infusions in patients with COVID-19 with moderate to severe ARDS (NCT04468971) or intubated and mechanically ventilated (NCT04482699).

Besides T_regs_, other T cell therapies are being evaluated for COVID-19 (Table 1). Viral-specific T cells are currently under investigation in three trials, and they are using viral-specific T cells from healthy donors who have mounted an appropriate response to the SARS-CoV-2 (NCT04457726, NCT04406064, and NCT04401410). A better understanding of effective targets could aid in the development of engineered T cells from more accessible and scalable sources than previously infected healthy donors. In addition, a phase 1/2 trial evaluating the use of RAPA-501, a hybrid T helper 2/T_reg_ phenotype, aims to suppress immune overactivation in a T cell receptor–independent manner (NCT04482699). Engineered T cells, particularly CAR T therapies, have shown promise in the treatment of immune system overactivation in diseases such as pemphigus vulgaris, type 1 diabetes, and lupus (*70*), and targeted T cell therapies could play a role in treating COVID-19 immune overactivation and facilitating viral clearance. Recent single-cell sequencing studies of patients with COVID-19 have shown an increase in monocytes, macrophages, and clonally expanded CD8^+^ T cells, which may contribute to the cytokine storm seen in severe cases (*71*, *72*). This provides a rationale to direct cell therapies such as CAR T/NK cells to target these enriched populations with the goal of reducing the excess cell population, and potentially decreasing the severity of the cytokine storm. In addition, B lymphocytes could theoretically be engineered to recombinantly express humanized monoclonal antibodies with neutralizing anti–SARS-CoV-2 activity. However, convalescent plasma or monoclonal antibodies likely have similar benefits without the increased complexity of a cell therapy–based modality (*73*).

In addition to their role in targeting COVID-19–related lung damage, MSCs are also an intriguing target for immune-based cell therapy because of their immunomodulatory capacities. In the lung, MSCs mediate immune homeostasis by TNF-α– and IL-1–induced up-regulation of anti-inflammatory cytokines such as protein TNF-stimulated gene 6, IL-10, TGF-β, prostaglandin E2, and nitric oxide (*74*, *75*). Moreover, by modulating overactivation of the immune system, MSCs have shown efficacy for the treatment of immune-related conditions such as steroid-refractory graft-versus-host disease and systemic lupus erythematosus (*76*, *77*). Hence, MSC therapy may play a role in suppressing COVID-19–associated immune activation and cytokine storm. Several recent studies have reported decreases in inflammatory marker levels after treatment with MSCs that correlated with clinical improvement (*41*–*44*). Moreover, ongoing clinical trials are assessing the immunomodulatory capabilities of MSCs in patients with COVID-19 (NCT04348435, NCT04377334, and NCT04397796). Another phase 1 clinical trial is assessing the efficacy and safety of allogeneic umbilical cord blood–derived MSCs as adjuvant therapy in patients receiving oseltamivir and azithromycin (NCT04457609). Dosing for MSC trials varies widely between 5 × 10^5^ and 1 × 10^7^ cells/kg or 2 × 10^7^ and 2 × 10^8^ cells per dose with the number of doses ranging from one to four. Cell sourcing for MSC trials includes the umbilical cord, placenta, adipose tissue, intra-aortic tissue, olfactory mucosa, and the dental pulp (*78*). More detailed reviews on mechanisms of MSC immunomodulation and potential benefits in COVID-19 have been previously explored (*75*, *78*–*89*).

## NEUROLOGICAL

Neurological manifestations are a significant consideration in patients with COVID-19 and are reported in 57.4% of confirmed cases (*90*). Presenting symptoms range from headache, anosmia, and ageusia to more serious manifestations such as ischemic stroke, encephalitis, and encephalomyelitis (*91*). The innate immune response is likely responsible for symptoms such as headache and encephalitis through uncontrolled cytokine release. However, symptoms such as anosmia, encephalomyelitis, and stroke suggest potential viral invasion of the central nervous system (CNS). Proposed mechanisms of CNS viral access include retrograde axonal transport through vagal afferents peripherally (*92*) or via direct CNS invasion, as studies have shown ACE2 receptors to be expressed in several regions of the brain, especially in oligodendrocytes and astrocytes (*93*). The symptoms of anosmia and ageusia were initially suggestive of CNS invasion, especially as SARS-COV studies had shown that the virus could enter the brain through the olfactory nerve within days of infection, causing inflammation and demyelination (*94*). However, analysis of human RNA sequencing and single-cell sequencing data showed that ACE2 and TMPRSS2 are not expressed in olfactory sensory nerves but instead in olfactory epithelium (*95*). Acute cerebral ischemic events have been reported in patients with COVID-19, especially in younger patients without typical risks of cerebrovascular disease (*96*, *97*). These manifestations are likely due to an overall prothrombotic state, potential down-regulation of ACE2, which causes an overall loss of neuroprotection, and hyperinflammatory cytokine release. In addition, there has been an increasing number of reports of Guillain-Barre syndrome and its variants, transverse myelitis, and other demyelinating conditions in affected patients, some with multifocal lesions in the brain and spine (*98*). The presence of demyelination has also been present in autopsy studies (*98*). The etiology of these lesions is likely immune-related, potentially because of a delayed immune reaction.

To date, there have been no reports of cell-based clinical trials addressing neurologic manifestations in patients with COVID-19. However, the high incidence of neurologic manifestations coupled with increasing reports of demyelinating disease and ischemic stroke in affected patients may require treatment options that focus on long-term deficits, which can potentially be addressed via cell therapy. Regarding demyelination, oligodendrocyte precursor cells (OPCs) have been explored in the setting of spinal cord injury and have showed safety, tolerability, cell engraftment, and improved motor function at 12-month follow-up in patients (NCT02302157). In addition, human iPSC (hiPSC)–derived OPCs were shown to remyelinate denuded axons in nonhuman primates with experimental autoimmune encephalomyelitis (EAE), a common animal model for multiple sclerosis (*99*). As COVID-19–related demyelination is likely due to immune-mediated myelin damage, successful applications of OPCs in other demyelinating animal models such as EAE suggest a potential benefit of OPCs in COVID-19–related refractory demyelination.

Patients with COVID-19 who suffered acute ischemic strokes, especially those with persistent deficits after mechanical thrombectomy or thrombolytic therapy, could also be a target of cell therapy. The long-term outcomes of patients suffering strokes, most of whom are younger and suffer large vessel occlusions, could be devastating. The prospect of stem cell therapies in stroke has expanded, with several concluded and ongoing clinical trials using bone marrow–derived stem cells and neural stem cells (*100*). MASTERS-2 (NCT03545607) and TREASURE (NCT02961504) are ongoing phase 3 clinical trials assessing treatment outcomes after intravenous administration of bone marrow–derived adult progenitor stem cells in patients suffering from ischemic stroke in the acute setting. Hence, this subpopulation of patients with COVID-19 may benefit from neuroregenerative cell therapies in the future.

## CARDIAC SYSTEM

Cardiac manifestations, such as elevated troponin levels and myocardial ischemic infarctions, are commonly seen in patients with COVID-19, particularly in severe presentations (*101*). Myocardial injury was found in 22% of hospitalized patients and nearly 60% of deceased patients (*4*, *14*). Moreover, cardiac arrhythmias were shown to be present in 44% of patients with COVID-19 in the ICU (*102*). Although cardiac cells express high levels of ACE2 (*11*), it remains unclear whether these cases constitute direct or indirect injury. One study on hiPSC-derived cardiomyocytes from patients with COVID-19 suggests viral invasion and cytopathic features in cardiac tissue (*103*). As cell therapies are designed, one potential way to mitigate the risk of SARS-CoV-2 viral entry of the treatment may be to genetically modulate viral entry proteins within the cell therapy itself. Indirect injury could be due to systemic hypoxia, secondary pulmonary hypertension, arrhythmia due to metabolic derangements, and cytokine storm damage (*104*).

Early cell therapy trials in acute myocardial infarct have largely focused on bone marrow mononuclear cells (BMMNCs), and early studies such as BOOST (*105*) and TOPCARE-MI (*106*) showed improvements in infarct size and left ventricular ejection fraction. Subsequent trials such as BOOST-2 (*107*) and TIME (*108*) showed no clinical benefit, however, questioning the role of BMMNCs in acute myocardial infarction. Preclinical data using a combination of cardiopoietic stem cells and MSCs have been promising and are under investigation in human trials (NCT02501811) (*109*). Further, Menasché *et al.* (*110*) showed that hESC-derived cardiac progenitors given to six patients with ischemic left ventricular dysfunction showed clinical improvement in systolic function without new tumors or arrhythmias. Clinical applications of iPSC-derived cardiomyocytes are also being explored (*111*). These advances in cell-based cardiac therapy can potentially be exploited for patients suffering from COVID-19–related cardiac ischemia. In addition, a recent clinical study used cardiosphere-derived cells, which are cardiac progenitor cells, to assess treatment of severe pulmonary manifestations in six patients with COVID-19. Four of the six patients were discharged from the hospital, while the remaining two were in stable conditions at the time the study was published (*112*). A phase 2 trial further assessing the efficacy of these cardiosphere-derived cells is currently under investigation (NCT04623671).

## GASTROINTESTINAL SYSTEM

Gastrointestinal manifestations occur in 5 to 10% of COVID-19 cases; however, symptoms have been mild and self-limited to nausea, diarrhea, and vomiting, despite ACE2 and TMPRSS2 being coexpressed in the small and large intestines and SARS-CoV-2 being detected in fecal samples of infected patients, suggesting direct viral invasion of enterocytes (*113*). This suggests that chronic intestinal sequela is unlikely to occur, negating the need for advanced treatments such as cell therapy. Hepatic involvement also appears to be frequent. Elevations in alanine aminotransferase and aspartate aminotransferase have been reported in up to a third of patients (*114*). ACE2 expression has been identified in cholangiocytes (*115*, *116*); however, histopathological examinations have yet to show direct viral inclusions in the liver (*117*). Other possibilities for hepatic injury may include immune-mediated damage, systemic hypoxia secondary to lung damage, and drug-induced liver injury (*118*). Stem cell–derived hepatic cells have been studied in the setting of acute and chronic liver failure. Patients have received cell therapies through the portal vein or splenic artery with improvement in serological markers such as prothrombin time or severity of hepatic encephalopathy (*119*). Although hepatocyte-based therapies have largely been considered a bridge to transplantation rather than a curative therapy itself, rare cases of patients with COVID-19 with acute liver failure may benefit from hepatocyte-based therapies (*120*).

## RENAL SYSTEM

Renal manifestations are frequent and range from mild proteinuria to severe injury requiring renal replacement therapy (*121*). Pei *et al.* (*122*) showed that 75% of patients with COVID-19 presenting with pneumonia were found to have an abnormal urine dipstick. Moreover, the presence of acute kidney injury (AKI) was associated with increased mortality, and only 46% of those patients who developed an AKI showed complete resolution after 12 days of follow-up. Over 80% of AKIs were intrinsic, with the remainder being secondary to rhabdomyolysis; there were no cases of prerenal AKI (*122*). Pathological studies have demonstrated acute tubular necrosis, presence of microthrombi, and mild interstitial fibrosis; however, no evidence of lymphocytic infiltrate in affected patients was found (*123*). While direct viral invasion is possible as ACE2 expression is present in tubular epithelium and podocytes, secondary mechanisms appear more relevant in inducing renal damage, which may include systemic hypoxia, rhabdomyolysis, cytokine-mediated damage, microemboli due to hypercoagulability, and cardiorenal congestion from right heart strain (*121*).

Cellular therapies for kidney disease are currently being explored and may benefit patients with COVID-19 suffering from permanent kidney injury. For example, preclinical studies using iPSC-derived renal precursor cells have shown the ability for these cells to engraft into damaged tubules and improve renal function (*124*). In addition, Swaminathan *et al.* (*125*) conducted a phase 2 trial using intra-aortic allogenic MSCs in the setting of postcardiac surgery–related AKI. However, the results showed no significant improvement in time to recover from AKI, dialysis use, or 30-day mortality. A phase 1 clinical trial, which is not yet recruiting, is aiming to assess the efficacy and safety of allogeneic MSCs infused via continuous renal replacement therapy (CRRT) in patients with COVID-19 with AKI undergoing CRRT (NCT04445220). Patients will be divided into three arms: low dose (2.5 × 10^7^ cells), high dose (7.5 × 10^7^ cells), and control. These studies could shed light on a possible role for cell therapies for the treatment of COVID-related renal damage.

## HEMATOLOGICAL SYSTEM

Hematological and vascular sequela, especially hypercoagulability and disseminated intravascular coagulation (DIC), are serious manifestations of SARS-CoV-2 (*126*). The hypercoagulable state increases the risk of venous thromboembolism, which can lead to ischemic stroke and multisystem organ failure via microemboli (*127*). Rates of venous thromboembolism in critically ill patients with COVID-19 have been estimated to be as high as 31% (*128*). Moreover, Tang *et al.* (*129*) reported that 70% of deceased patients met criteria for DIC. The hypercoagulable state may be related to stimulated production of antiphospholipid antibodies and complement activation, vascular endothelial damage, and prolonged immobility in the ICU (*130*). Although the hypercoagulable state is likely due to a variety of factors, endothelial disruption is one potential cause that may contribute to multisystem end-organ damage in COVID-19 (*131*). CD34^+^ cells, hematopoietic stem cells that can give rise to endothelial progenitors and restore vasculature, have been approved for an investigational new drug by the FDA to assess their efficacy and safety for lung damage repair. CD34^+^ cells are thought to promote vascular regeneration to counter ischemic damage and have shown efficacy and safety in trials evaluating their potential in cardiac and critical limb ischemia (*132*). Cord blood CD34^+^ cells also showed protective effects on acute lung injury induced by lipopolysaccharide challenge in mice, similar to another study that showed that peripheral blood CD34^+^ cells attenuated acute lung injury induced by oleic acid in rats (*133*, *134*). Hence, therapy with CD34^+^ cells could prove feasible for promoting vascular growth in the lungs of patients with COVID-19 suffering from significant pulmonary damage (NCT04522817).

## FUTURE DIRECTIONS AND LIMITATIONS

Overall, cell therapies show great promise in several diseases, and data from other studies suggest that certain cell therapies may be applicable in particular pathogenesis aspects of COVID-19. Specific factors such as dosing of the cells, route of administration, allogenic versus autologous cells, role of immunosuppressive therapy, tolerance of treatment in elderly patients, role of extracellular vesicles, and readouts of effectiveness need to be better delineated. As an example, risk for severe illness with COVID-19 increases with age. There are lessons to be learned about recipient age from studies using hematopoietic stem cell transplantation (HSCT) or MSCs. For instance, HSCT studies have shown that patient age is correlated with transplant-related morbidity and mortality, but improvements such as the use of cytokines and less toxic or reduced conditioning have allowed older patients to receive these therapies. In the context of MSCs, a study conducted to evaluate patient age on the efficacy of MSC cell therapy in ischemic cardiomyopathy showed that older patients did not have an impaired response. Although these studies are not directly translatable to other cell types or patients with COVID-19, they nevertheless represent a starting point for future investigation (*135*–*140*). Cell dosing and number of injections should be tailored to patient-specific responses and tolerance of treatment. Route of administration should be localized as much as possible to reduce the risk of unintentional side effects in distant organs while maximizing efficacy at the infected organ system. Disseminated coronavirus involving multiple organ systems, for example, may benefit from intravenous infusion of cell therapy to allow treatment to reach multiple infected organs. Various routes of administration have been previously explored for respiratory and pulmonary diseases including intravenous, intratracheal instillation, inhalation, aerosolization, and nebulizers. Intratracheal instillation could be advantageous, as it provides highly precise, local delivery to the respiratory tract using a small dose; however, instillation is highly nonphysiological and may result in inconsistent and heterogeneous deposition focused on the upper airways (*141*). Five clinical trials for lung cell therapies have used aerosolization as the route of administration (NCT04313647, NCT04473170, NCT04389385, NCT04491240, and NCT04276987). This route of administration may be preferred because of the potentially broader distribution of cells in the lung while reducing the probability of cell damage and loss (*141*).

Another interesting avenue to consider is the use of a combination of various cell therapies. MSCs, for example, have been studied for their synergistic effects with other cell types, including pulmonary endothelial cells and epithelial cells (*142*). For instance, MSCs were shown to stimulate endothelial progenitors in patients with heart failure and preserve endothelial integrity after hemorrhagic shock (*143*, *144*). These findings could support investigation of both cell types as a combination cell therapy.

From a scalability standpoint, allogenic or off-the-shelf–based therapies that are either human leukocyte antigen (HLA)–matched or do not have HLAs present would be favored over autologous cells. HLA matching or depletion may also reduce the need for immunosuppression. Clinical trial readouts should include COVID-19–related outcomes and organ function related to the cell therapy being administered. Last, the idea of leveraging the field of synthetic biology to further adapt engineered cell lines should also be considered. For example, cell therapies that modulate expression of viral entry proteins, decrease residual potentially tumorigenic pluripotent cells, or adopt genome-scale mammalian translational recoding to confer viral resistance could be of keen advantage (*145*, *146*).

## References

[1] Z. Wu, J. M. McGoogan, Characteristics of and important lessons from the coronavirus disease 2019 (COVID-19) outbreak in China: Summary of a report of 72314 cases from the Chinese Center for Disease Control and Prevention. JAMA 323, 1239–1242 (2020). 32091533 10.1001/jama.2020.2648

[2] X. Yang, Y. Yu, J. Xu, H. Shu, J. Xia, H. Liu, Y. Wu, L. Zhang, Z. Yu, M. Fang, T. Yu, Y. Wang, S. Pan, X. Zou, S. Yuan, Y. Shang, Clinical course and outcomes of critically ill patients with SARS-CoV-2 pneumonia in Wuhan, China: A single-centered, retrospective, observational study. Lancet Respir. Med. 8, 475–481 (2020). 32105632 10.1016/S2213-2600(20)30079-5 PMC7102538

[3] M. Hoffmann, H. Kleine-Weber, S. Schroeder, N. Kruger, T. Herrler, S. Erichsen, T. S. Schiergens, G. Herrler, N. H. Wu, A. Nitsche, M. A. Muller, C. Drosten, S. Pohlmann, SARS-CoV-2 cell entry depends on ACE2 and TMPRSS2 and is blocked by a clinically proven protease inhibitor. Cell 181, 271–280.e8 (2020). 32142651 10.1016/j.cell.2020.02.052 PMC7102627

[4] P. Zhou, X. L. Yang, X. G. Wang, B. Hu, L. Zhang, W. Zhang, H. R. Si, Y. Zhu, B. Li, C. L. Huang, H. D. Chen, J. Chen, Y. Luo, H. Guo, R. D. Jiang, M. Q. Liu, Y. Chen, X. R. Shen, X. Wang, X. S. Zheng, K. Zhao, Q. J. Chen, F. Deng, L. L. Liu, B. Yan, F. X. Zhan, Y. Y. Wang, G. F. Xiao, Z. L. Shi, A pneumonia outbreak associated with a new coronavirus of probable bat origin. Nature 579, 270–273 (2020). 32015507 10.1038/s41586-020-2012-7 PMC7095418

[5] D. E. Gordon, G. M. Jang, M. Bouhaddou, J. Xu, K. Obernier, K. M. White, M. J. O’Meara, V. V. Rezelj, J. Z. Guo, D. L. Swaney, T. A. Tummino, R. Huettenhain, R. M. Kaake, A. L. Richards, B. Tutuncuoglu, H. Foussard, J. Batra, K. Haas, M. Modak, M. Kim, P. Haas, B. J. Polacco, H. Braberg, J. M. Fabius, M. Eckhardt, M. Soucheray, M. J. Bennett, M. Cakir, M. J. McGregor, Q. Li, B. Meyer, F. Roesch, T. Vallet, A. M. Kain, L. Miorin, E. Moreno, Z. Z. C. Naing, Y. Zhou, S. Peng, Y. Shi, Z. Zhang, W. Shen, I. T. Kirby, J. E. Melnyk, J. S. Chorba, K. Lou, S. A. Dai, I. Barrio-Hernandez, D. Memon, C. Hernandez-Armenta, J. Lyu, C. J. P. Mathy, T. Perica, K. B. Pilla, S. J. Ganesan, D. J. Saltzberg, R. Rakesh, X. Liu, S. B. Rosenthal, L. Calviello, S. Venkataramanan, J. Liboy-Lugo, Y. Lin, X. P. Huang, Y. Liu, S. A. Wankowicz, M. Bohn, M. Safari, F. S. Ugur, C. Koh, N. S. Savar, Q. D. Tran, D. Shengjuler, S. J. Fletcher, M. C. O’Neal, Y. Cai, J. C. J. Chang, D. J. Broadhurst, S. Klippsten, P. P. Sharp, N. A. Wenzell, D. Kuzuoglu, H. Y. Wang, R. Trenker, J. M. Young, D. A. Cavero, J. Hiatt, T. L. Roth, U. Rathore, A. Subramanian, J. Noack, M. Hubert, R. M. Stroud, A. D. Frankel, O. S. Rosenberg, K. A. Verba, D. A. Agard, M. Ott, M. Emerman, N. Jura, M. von Zastrow, E. Verdin, A. Ashworth, O. Schwartz, C. d’Enfert, S. Mukherjee, M. Jacobson, H. S. Malik, D. G. Fujimori, T. Ideker, C. S. Craik, S. N. Floor, J. S. Fraser, J. D. Gross, A. Sali, B. L. Roth, D. Ruggero, J. Taunton, T. Kortemme, P. Beltrao, M. Vignuzzi, A. Garcia-Sastre, K. M. Shokat, B. K. Shoichet, N. J. Krogan, A SARS-CoV-2 protein interaction map reveals targets for drug repurposing. Nature 583, 459–468 (2020). 32353859 10.1038/s41586-020-2286-9 PMC7431030

[6] L. Cantuti-Castelvetri, R. Ojha, L. D. Pedro, M. Djannatian, J. Franz, S. Kuivanen, F. van der Meer, K. Kallio, T. Kaya, M. Anastasina, T. Smura, L. Levanov, L. Szirovicza, A. Tobi, H. Kallio-Kokko, P. Osterlund, M. Joensuu, F. A. Meunier, S. J. Butcher, M. S. Winkler, B. Mollenhauer, A. Helenius, O. Gokce, T. Teesalu, J. Hepojoki, O. Vapalahti, C. Stadelmann, G. Balistreri, M. Simons, Neuropilin-1 facilitates SARS-CoV-2 cell entry and infectivity. Science 370, 856–860 (2020). 33082293 10.1126/science.abd2985 PMC7857391

[7] J. L. Daly, B. Simonetti, K. Klein, K. E. Chen, M. K. Williamson, C. Anton-Plagaro, D. K. Shoemark, L. Simon-Gracia, M. Bauer, R. Hollandi, U. F. Greber, P. Horvath, R. B. Sessions, A. Helenius, J. A. Hiscox, T. Teesalu, D. A. Matthews, A. D. Davidson, B. M. Collins, P. J. Cullen, Y. Yamauchi, Neuropilin-1 is a host factor for SARS-CoV-2 infection. Science 370, 861–865 (2020). 33082294 10.1126/science.abd3072 PMC7612957

[8] S. Soni, Y. Jiang, Y. Tesfaigzi, J. L. Hornick, S. Cataltepe, Comparative analysis of ACE2 protein expression in rodent, non-human primate, and human respiratory tract at baseline and after injury: A conundrum for COVID-19 pathogenesis. PLOS ONE 16, e0247510 (2021). 33626084 10.1371/journal.pone.0247510 PMC7904186

[9] S. F. Masre, N. F. Jufri, F. W. Ibrahim, S. H. Abdul Raub, Classical and alternative receptors for SARS-CoV-2 therapeutic strategy. Rev. Med. Virol. e2207 (2020). 33368788 10.1002/rmv.2207 PMC7883063

[10] J. C. Keen, H. M. Moore, The Genotype-Tissue Expression (GTEx) Project: Linking clinical data with molecular analysis to advance personalized medicine. J. Pers. Med. 5, 22–29 (2015). 25809799 10.3390/jpm5010022 PMC4384056

[11] M. Uhlén, L. Fagerberg, B. M. Hallström, C. Lindskog, P. Oksvold, A. Mardinoglu, A. Sivertsson, C. Kampf, E. Sjostedt, A. Asplund, I. Olsson, K. Edlund, E. Lundberg, S. Navani, C. A. Szigyarto, J. Odeberg, D. Djureinovic, J. O. Takanen, S. Hober, T. Alm, P. H. Edqvist, H. Berling, H. Tegel, J. Mulder, J. Rockberg, P. Nilsson, J. M. Schwenk, M. Hamsten, K. von Feilitzen, M. Forsberg, L. Persson, F. Johansson, M. Zwahlen, G. von Heijne, J. Nielsen, F. Pontén, Proteomics. Tissue-based map of the human proteome. Science 347, 1260419 (2015). 25613900 10.1126/science.1260419

[12] M. Singh, V. Bansal, C. Feschotte, A single-cell RNA expression map of human coronavirus entry factors. Cell Rep. 32, 108175 (2020). 32946807 10.1016/j.celrep.2020.108175 PMC7470764

[13] Y. J. Hou, K. Okuda, C. E. Edwards, D. R. Martinez, T. Asakura, K. H. Dinnon III, T. Kato, R. E. Lee, B. L. Yount, T. M. Mascenik, G. Chen, K. N. Olivier, A. Ghio, L. V. Tse, S. R. Leist, L. E. Gralinski, A. Schafer, H. Dang, R. Gilmore, S. Nakano, L. Sun, M. L. Fulcher, A. Livraghi-Butrico, N. I. Nicely, M. Cameron, C. Cameron, D. J. Kelvin, A. de Silva, D. M. Margolis, A. Markmann, L. Bartelt, R. Zumwalt, F. J. Martinez, S. P. Salvatore, A. Borczuk, P. R. Tata, V. Sontake, A. Kimple, I. Jaspers, W. K. O’Neal, S. H. Randell, R. C. Boucher, R. S. Baric, SARS-CoV-2 reverse genetics reveals a variable infection gradient in the respiratory tract. Cell 182, 429–446.e14 (2020). 32526206 10.1016/j.cell.2020.05.042 PMC7250779

[14] F. Zhou, T. Yu, R. Du, G. Fan, Y. Liu, Z. Liu, J. Xiang, Y. Wang, B. Song, X. Gu, L. Guan, Y. Wei, H. Li, X. Wu, J. Xu, S. Tu, Y. Zhang, H. Chen, B. Cao, Clinical course and risk factors for mortality of adult inpatients with COVID-19 in Wuhan, China: A retrospective cohort study. Lancet 395, 1054–1062 (2020). 32171076 10.1016/S0140-6736(20)30566-3 PMC7270627

[15] Z. Imam, F. Odish, I. Gill, D. O’Connor, J. Armstrong, A. Vanood, O. Ibironke, A. Hanna, A. Ranski, A. Halalau, Older age and comorbidity are independent mortality predictors in a large cohort of 1305 COVID-19 patients in Michigan, United States. J. Intern. Med. 288, 469–476 (2020). 32498135 10.1111/joim.13119 PMC7300881

[16] N. Mehta, M. Mazer-Amirshahi, N. Alkindi, A. Pourmand, Pharmacotherapy in COVID-19; A narrative review for emergency providers. Am. J. Emerg. Med. 38, 1488–1493 (2020). 32336586 10.1016/j.ajem.2020.04.035 PMC7158837

[17] S. Feins, W. Kong, E. F. Williams, M. C. Milone, J. A. Fraietta, An introduction to chimeric antigen receptor (CAR) T-cell immunotherapy for human cancer. Am. J. Hematol. 94, S3–S9 (2019). 10.1002/ajh.25418 30680780

[18] C. H. June, R. S. O’Connor, O. U. Kawalekar, S. Ghassemi, M. C. Milone, CAR T cell immunotherapy for human cancer. Science 359, 1361–1365 (2018). 29567707 10.1126/science.aar6711

[19] B. Parekkadan, J. M. Milwid, Mesenchymal stem cells as therapeutics. Annu. Rev. Biomed. Eng. 12, 87–117 (2010). 20415588 10.1146/annurev-bioeng-070909-105309 PMC3759519

[20] V. K. Singh, M. Kalsan, N. Kumar, A. Saini, R. Chandra, Induced pluripotent stem cells: Applications in regenerative medicine, disease modeling, and drug discovery. Front. Cell Dev. Biol. 3, 2 (2015). 25699255 10.3389/fcell.2015.00002 PMC4313779

[21] J. A. Ankrum, J. F. Ong, J. M. Karp, Mesenchymal stem cells: Immune evasive, not immune privileged. Nat. Biotechnol. 32, 252–260 (2014). 24561556 10.1038/nbt.2816 PMC4320647

[22] J. M. Ryan, F. P. Barry, J. M. Murphy, B. P. Mahon, Mesenchymal stem cells avoid allogeneic rejection. J. Inflamm. 2, 8 (2005). 10.1186/1476-9255-2-8 PMC1215510 16045800

[23] T. Deuse, X. Hu, A. Gravina, D. Wang, G. Tediashvili, C. De, W. O. Thayer, A. Wahl, J. V. Garcia, H. Reichenspurner, M. M. Davis, L. L. Lanier, S. Schrepfer, Hypoimmunogenic derivatives of induced pluripotent stem cells evade immune rejection in fully immunocompetent allogeneic recipients. Nat. Biotechnol. 37, 252–258 (2019). 30778232 10.1038/s41587-019-0016-3 PMC6419516

[24] X. Han, M. Wang, S. Duan, P. J. Franco, J. H. Kenty, P. Hedrick, Y. Xia, A. Allen, L. M. R. Ferreira, J. L. Strominger, D. A. Melton, T. B. Meissner, C. A. Cowan, Generation of hypoimmunogenic human pluripotent stem cells. Proc. Natl. Acad. Sci. U.S.A. 116, 10441–10446 (2019). 31040209 10.1073/pnas.1902566116 PMC6535035

[25] L. Barkholt, E. Flory, V. Jekerle, S. Lucas-Samuel, P. Ahnert, L. Bisset, D. Buscher, W. Fibbe, A. Foussat, M. Kwa, O. Lantz, R. Maciulaitis, T. Palomaki, C. K. Schneider, L. Sensebe, G. Tachdjian, K. Tarte, L. Tosca, P. Salmikangas, Risk of tumorigenicity in mesenchymal stromal cell-based therapies--bridging scientific observations and regulatory viewpoints. Cytotherapy 15, 753–759 (2013). 23602595 10.1016/j.jcyt.2013.03.005

[26] U. Ben-David, N. Benvenisty, The tumorigenicity of human embryonic and induced pluripotent stem cells. Nat. Rev. Cancer 11, 268–277 (2011). 21390058 10.1038/nrc3034

[27] Y. Sato, H. Bando, M. Di Piazza, G. Gowing, C. Herberts, S. Jackman, G. Leoni, S. Libertini, T. MacLachlan, J. W. McBlane, L. Pereira Mouries, M. Sharpe, W. Shingleton, B. Surmacz-Cordle, K. Yamamoto, J. W. van der Laan, Tumorigenicity assessment of cell therapy products: The need for global consensus and points to consider. Cytotherapy 21, 1095–1111 (2019). 31711733 10.1016/j.jcyt.2019.10.001

[28] U. Martin, Therapeutic application of pluripotent stem cells: Challenges and risks. Front. Med. 4, 229 (2017). 10.3389/fmed.2017.00229 PMC5735065 29312943

[29] K. Wang, R. Z. Lin, X. Hong, A. H. Ng, C. N. Lee, J. Neumeyer, G. Wang, X. Wang, M. Ma, W. T. Pu, G. M. Church, J. M. Melero-Martin, Robust differentiation of human pluripotent stem cells into endothelial cells via temporal modulation of ETV2 with modified mRNA. Sci. Adv. 6, eaba7606 (2020). 32832668 10.1126/sciadv.aba7606 PMC7439318

[30] V. Busskamp, N. E. Lewis, P. Guye, A. H. Ng, S. L. Shipman, S. M. Byrne, N. E. Sanjana, J. Murn, Y. Li, S. Li, M. Stadler, R. Weiss, G. M. Church, Rapid neurogenesis through transcriptional activation in human stem cells. Mol. Syst. Biol. 10, 760 (2014). 25403753 10.15252/msb.20145508 PMC4299601

[31] G. Itakura, S. Kawabata, M. Ando, Y. Nishiyama, K. Sugai, M. Ozaki, T. Iida, T. Ookubo, K. Kojima, R. Kashiwagi, K. Yasutake, H. Nakauchi, H. Miyoshi, N. Nagoshi, J. Kohyama, A. Iwanami, M. Matsumoto, M. Nakamura, H. Okano, Fail-safe system against potential tumorigenicity after transplantation of iPSC derivatives. Stem Cell Rep. 8, 673–684 (2017). 10.1016/j.stemcr.2017.02.003 PMC5355810 28262544

[32] C. Huang, Y. Wang, X. Li, L. Ren, J. Zhao, Y. Hu, L. Zhang, G. Fan, J. Xu, X. Gu, Z. Cheng, T. Yu, J. Xia, Y. Wei, W. Wu, X. Xie, W. Yin, H. Li, M. Liu, Y. Xiao, H. Gao, L. Guo, J. Xie, G. Wang, R. Jiang, Z. Gao, Q. Jin, J. Wang, B. Cao, Clinical features of patients infected with 2019 novel coronavirus in Wuhan, China. Lancet 395, 497–506 (2020). 31986264 10.1016/S0140-6736(20)30183-5 PMC7159299

[33] D. Huang, X. Lian, F. Song, H. Ma, Z. Lian, Y. Liang, T. Qin, W. Chen, S. Wang, Clinical features of severe patients infected with 2019 novel coronavirus: A systematic review and meta-analysis. Ann. Transl. Med. 8, 576 (2020). 32566603 10.21037/atm-20-2124 PMC7290556

[34] W. Zhang, Imaging changes of severe COVID-19 pneumonia in advanced stage. Intensive Care Med. 46, 841–843 (2020). 32123986 10.1007/s00134-020-05990-y PMC7080018

[35] D. McGonagle, J. S. O’Donnell, K. Sharif, P. Emery, C. Bridgewood, Immune mechanisms of pulmonary intravascular coagulopathy in COVID-19 pneumonia. Lancet Rheumatol. 2, e437–e445 (2020). 32835247 10.1016/S2665-9913(20)30121-1 PMC7252093

[36] P. M. George, A. U. Wells, R. G. Jenkins, Pulmonary fibrosis and COVID-19: The potential role for antifibrotic therapy. Lancet Respir. Med. 8, 807–815 (2020). 32422178 10.1016/S2213-2600(20)30225-3 PMC7228727

[37] C. R. Harrell, R. Sadikot, J. Pascual, C. Fellabaum, M. G. Jankovic, N. Jovicic, V. Djonov, N. Arsenijevic, V. Volarevic, Mesenchymal stem cell-based therapy of inflammatory lung diseases: Current understanding and future perspectives. Stem Cells Int. 2019, 4236973 (2019). 31191672 10.1155/2019/4236973 PMC6525794

[38] M. C. Chan, D. I. Kuok, C. Y. Leung, K. P. Hui, S. A. Valkenburg, E. H. Lau, J. M. Nicholls, X. Fang, Y. Guan, J. W. Lee, R. W. Chan, R. G. Webster, M. A. Matthay, J. S. Peiris, Human mesenchymal stromal cells reduce influenza A H5N1-associated acute lung injury in vitro and in vivo. Proc. Natl. Acad. Sci. U.S.A. 113, 3621–3626 (2016). 26976597 10.1073/pnas.1601911113 PMC4822574

[39] Y. Li, J. Xu, W. Shi, C. Chen, Y. Shao, L. Zhu, W. Lu, X. Han, Mesenchymal stromal cell treatment prevents H9N2 avian influenza virus-induced acute lung injury in mice. Stem Cell Res. Ther. 7, 159 (2016). 27793190 10.1186/s13287-016-0395-z PMC5084318

[40] T. J. Morrison, M. V. Jackson, E. K. Cunningham, A. Kissenpfennig, D. F. McAuley, C. M. O’Kane, A. D. Krasnodembskaya, Mesenchymal stromal cells modulate macrophages in clinically relevant lung injury models by extracellular vesicle mitochondrial transfer. Am. J. Respir. Crit. Care Med. 196, 1275–1286 (2017). 28598224 10.1164/rccm.201701-0170OC PMC5694830

[41] B. Liang, J. Chen, T. Li, H. Wu, W. Yang, Y. Li, J. Li, C. Yu, F. Nie, Z. Ma, M. Yang, M. Xiao, P. Nie, Y. Gao, C. Qian, M. Hu, Clinical remission of a critically ill COVID-19 patient treated by human umbilical cord mesenchymal stem cells: A case report. Medicine 99, e21429 (2020). 32756149 10.1097/MD.0000000000021429 PMC7402800

[42] L. Tang, Y. Jiang, M. Zhu, L. Chen, X. Zhou, C. Zhou, P. Ye, X. Chen, B. Wang, Z. Xu, Q. Zhang, X. Xu, H. Gao, X. Wu, D. Li, W. Jiang, J. Qu, C. Xiang, L. Li, Clinical study using mesenchymal stem cells for the treatment of patients with severe COVID-19. Front. Med. 14, 664–673 (2020). 32761491 10.1007/s11684-020-0810-9 PMC7406954

[43] Z. Leng, R. Zhu, W. Hou, Y. Feng, Y. Yang, Q. Han, G. Shan, F. Meng, D. Du, S. Wang, J. Fan, W. Wang, L. Deng, H. Shi, H. Li, Z. Hu, F. Zhang, J. Gao, H. Liu, X. Li, Y. Zhao, K. Yin, X. He, Z. Gao, Y. Wang, B. Yang, R. Jin, I. Stambler, L. W. Lim, H. Su, A. Moskalev, A. Cano, S. Chakrabarti, K. J. Min, G. Ellison-Hughes, C. Caruso, K. Jin, R. C. Zhao, Transplantation of ACE2^−^ mesenchymal stem cells improves the outcome of patients with COVID-19 pneumonia. Aging Dis. 11, 216–228 (2020). 32257537 10.14336/AD.2020.0228 PMC7069465

[44] F. Sanchez-Guijo, M. García-Arranz, M. López-Parra, P. Monedero, C. Mata-Martínez, A. Santos, V. Sagredo, J.-M. Álvarez-Avello, J. E. Guerrero, C. Pérez-Calvo, M.-V. Sánchez-Hernández, J. L. Del-Pozo, E. J. Andreu, M.-E. Fernández-Santos, B. Soria-Juan, L. M. Hernández-Blasco, E. Andreu, J. M. Sempere, A. G. Zapata, J. M. Moraleda, B. Soria, F. Fernández-Avilés, D. García-Olmo, F. Prósper, Adipose-derived mesenchymal stromal cells for the treatment of patients with severe SARS-CoV-2 pneumonia requiring mechanical ventilation. A proof of concept study. EClinicalMedicine 25, 100454 (2020). 32838232 10.1016/j.eclinm.2020.100454 PMC7348610

[45] G. Lanzoni, E. Linetsky, D. Correa, S. M. Cayetano, R. A. Alvarez, D. Kouroupis, A. Alvarez Gil, R. Poggioli, P. Ruiz, A. C. Marttos, K. Hirani, C. A. Bell, H. Kusack, L. Rafkin, D. Baidal, A. Pastewski, K. Gawri, C. Lenero, A. M. A. Mantero, S. W. Metalonis, X. Wang, L. Roque, B. Masters, N. S. Kenyon, E. Ginzburg, X. Xu, J. Tan, A. I. Caplan, M. K. Glassberg, R. Alejandro, C. Ricordi, Umbilical cord mesenchymal stem cells for COVID-19 acute respiratory distress syndrome: A double-blind, phase 1/2a, randomized controlled trial. Stem Cells Transl. Med. 10, 660–673 (2021). 33400390 10.1002/sctm.20-0472 PMC8046040

[46] F. Grillo, E. Barisione, L. Ball, L. Mastracci, R. Fiocca, Lung fibrosis: An undervalued finding in COVID-19 pathological series. Lancet Infect. Dis. 21, e72 (2021). 32735785 10.1016/S1473-3099(20)30582-X PMC7386839

[47] R. Laporta Hernandez, M. Aguilar Perez, M. T. Lazaro Carrasco, P. Ussetti Gil, Lung transplantation in idiopathic pulmonary fibrosis. Med. Sci. 6, 68 (2018). 10.3390/medsci6030068 PMC6164271 30142942

[48] A. Serrano-Mollar, M. Nacher, G. Gay-Jordi, D. Closa, A. Xaubet, O. Bulbena, Intratracheal transplantation of alveolar type II cells reverses bleomycin-induced lung fibrosis. Am. J. Respir. Crit. Care Med. 176, 1261–1268 (2007). 17641155 10.1164/rccm.200610-1491OC

[49] D. Wang, J. E. Morales, D. G. Calame, J. L. Alcorn, R. A. Wetsel, Transplantation of human embryonic stem cell-derived alveolar epithelial type II cells abrogates acute lung injury in mice. Mol. Ther. 18, 625–634 (2010). 20087316 10.1038/mt.2009.317 PMC2839438

[50] A. Serrano-Mollar, G. Gay-Jordi, R. Guillamat-Prats, D. Closa, F. Hernandez-Gonzalez, P. Marin, F. Burgos, J. Martorell, M. Sanchez, P. Arguis, D. Soy, J. M. Bayas, J. Ramirez, T. D. Tetley, L. Molins, J. P. de la Bellacasa, C. Rodriguez-Villar, I. Rovira, J. J. Fibla, A. Xaubet; Pneumocyte Study Group, Safety and tolerability of alveolar type II cell transplantation in idiopathic pulmonary fibrosis. Chest 150, 533–543 (2016). 27020420 10.1016/j.chest.2016.03.021

[51] A. Averyanov, I. Koroleva, M. Konoplyannikov, V. Revkova, V. Lesnyak, V. Kalsin, O. Danilevskaya, A. Nikitin, A. Sotnikova, S. Kotova, V. Baklaushev, First-in-human high-cumulative-dose stem cell therapy in idiopathic pulmonary fibrosis with rapid lung function decline. Stem Cells Transl. Med. 9, 6–16 (2020). 31613055 10.1002/sctm.19-0037 PMC6954714

[52] D. F. Robbiani, C. Gaebler, F. Muecksch, J. C. C. Lorenzi, Z. Wang, A. Cho, M. Agudelo, C. O. Barnes, A. Gazumyan, S. Finkin, T. Hagglof, T. Y. Oliveira, C. Viant, A. Hurley, H. H. Hoffmann, K. G. Millard, R. G. Kost, M. Cipolla, K. Gordon, F. Bianchini, S. T. Chen, V. Ramos, R. Patel, J. Dizon, I. Shimeliovich, P. Mendoza, H. Hartweger, L. Nogueira, M. Pack, J. Horowitz, F. Schmidt, Y. Weisblum, E. Michailidis, A. W. Ashbrook, E. Waltari, J. E. Pak, K. E. Huey-Tubman, N. Koranda, P. R. Hoffman, A. P. West Jr., C. M. Rice, T. Hatziioannou, P. J. Bjorkman, P. D. Bieniasz, M. Caskey, M. C. Nussenzweig, Convergent antibody responses to SARS-CoV-2 in convalescent individuals. Nature 584, 437–442 (2020). 32555388 10.1038/s41586-020-2456-9 PMC7442695

[53] C. M. Poh, G. Carissimo, B. Wang, S. N. Amrun, C. Y. Lee, R. S. Chee, S. W. Fong, N. K. Yeo, W. H. Lee, A. Torres-Ruesta, Y. S. Leo, M. I. Chen, S. Y. Tan, L. Y. A. Chai, S. Kalimuddin, S. S. G. Kheng, S. Y. Thien, B. E. Young, D. C. Lye, B. J. Hanson, C. I. Wang, L. Renia, L. F. P. Ng, Two linear epitopes on the SARS-CoV-2 spike protein that elicit neutralising antibodies in COVID-19 patients. Nat. Commun. 11, 2806 (2020). 32483236 10.1038/s41467-020-16638-2 PMC7264175

[54] J. Zhao, Q. Yuan, H. Wang, W. Liu, X. Liao, Y. Su, X. Wang, J. Yuan, T. Li, J. Li, S. Qian, C. Hong, F. Wang, Y. Liu, Z. Wang, Q. He, Z. Li, B. He, T. Zhang, Y. Fu, S. Ge, L. Liu, J. Zhang, N. Xia, Z. Zhang, Antibody responses to SARS-CoV-2 in patients with novel coronavirus disease 2019. Clin. Infect. Dis. 71, 2027–2034 (2020). 32221519 10.1093/cid/ciaa344 PMC7184337

[55] A. Iwasaki, Y. Yang, The potential danger of suboptimal antibody responses in COVID-19. Nat. Rev. Immunol. 20, 339–341 (2020). 32317716 10.1038/s41577-020-0321-6 PMC7187142

[56] N. Eroshenko, T. Gill, M. K. Keaveney, G. M. Church, J. M. Trevejo, H. Rajaniemi, Implications of antibody-dependent enhancement of infection for SARS-CoV-2 countermeasures. Nat. Biotechnol. 38, 789–791 (2020). 32504046 10.1038/s41587-020-0577-1

[57] B. Diao, C. Wang, Y. Tan, X. Chen, Y. Liu, L. Ning, L. Chen, M. Li, Y. Liu, G. Wang, Z. Yuan, Z. Feng, Y. Zhang, Y. Wu, Y. Chen, Reduction and functional exhaustion of T cells in patients with coronavirus disease 2019 (COVID-19). Front. Immunol. 11, 827 (2020). 32425950 10.3389/fimmu.2020.00827 PMC7205903

[58] J. Mu, J. Xu, L. Zhang, T. Shu, D. Wu, M. Huang, Y. Ren, X. Li, Q. Geng, Y. Xu, Y. Qiu, X. Zhou, SARS-CoV-2-encoded nucleocapsid protein acts as a viral suppressor of RNA interference in cells. Sci. China Life Sci. 63, 1413–1416 (2020). 32291557 10.1007/s11427-020-1692-1 PMC7154568

[59] X. Lu, J. Pan, J. Tao, D. Guo, SARS-CoV nucleocapsid protein antagonizes IFN-β response by targeting initial step of IFN-β induction pathway, and its C-terminal region is critical for the antagonism. Virus Genes 42, 37–45 (2011). 20976535 10.1007/s11262-010-0544-x PMC7088804

[60] R. Channappanavar, S. Perlman, Pathogenic human coronavirus infections: Causes and consequences of cytokine storm and immunopathology. Semin. Immunopathol. 39, 529–539 (2017). 28466096 10.1007/s00281-017-0629-x PMC7079893

[61] I. Prager, C. Watzl, Mechanisms of natural killer cell-mediated cellular cytotoxicity. J. Leukoc. Biol. 105, 1319–1329 (2019). 31107565 10.1002/JLB.MR0718-269R

[62] E. Liu, D. Marin, P. Banerjee, H. A. Macapinlac, P. Thompson, R. Basar, L. Nassif Kerbauy, B. Overman, P. Thall, M. Kaplan, V. Nandivada, I. Kaur, A. Nunez Cortes, K. Cao, M. Daher, C. Hosing, E. N. Cohen, P. Kebriaei, R. Mehta, S. Neelapu, Y. Nieto, M. Wang, W. Wierda, M. Keating, R. Champlin, E. J. Shpall, K. Rezvani, Use of CAR-transduced natural killer cells in CD19-positive lymphoid tumors. N. Engl. J. Med. 382, 545–553 (2020). 32023374 10.1056/NEJMoa1910607 PMC7101242

[63] S. Schmidt, L. Tramsen, B. Rais, E. Ullrich, T. Lehrnbecher, Natural killer cells as a therapeutic tool for infectious diseases - current status and future perspectives. Oncotarget 9, 20891–20907 (2018). 29755697 10.18632/oncotarget.25058 PMC5945539

[64] M. Market, L. Angka, A. B. Martel, D. Bastin, O. Olanubi, G. Tennakoon, D. M. Boucher, J. Ng, M. Ardolino, R. C. Auer, Flattening the COVID-19 curve with natural killer cell based immunotherapies. Front. Immunol. 11, 1512 (2020). 32655581 10.3389/fimmu.2020.01512 PMC7324763

[65] L. M. R. Ferreira, Y. D. Muller, J. A. Bluestone, Q. Tang, Next-generation regulatory T cell therapy. Nat. Rev. Drug Discov. 18, 749–769 (2019). 31541224 10.1038/s41573-019-0041-4 PMC7773144

[66] E. B. Okeke, J. E. Uzonna, The pivotal role of regulatory T cells in the regulation of innate immune cells. Front. Immunol. 10, 680 (2019). 31024539 10.3389/fimmu.2019.00680 PMC6465517

[67] M. Romano, G. Fanelli, C. J. Albany, G. Giganti, G. Lombardi, Past, present, and future of regulatory T cell therapy in transplantation and autoimmunity. Front. Immunol. 10, 43 (2019). 30804926 10.3389/fimmu.2019.00043 PMC6371029

[68] X. Zhang, N. Olsen, S. G. Zheng, The progress and prospect of regulatory T cells in autoimmune diseases. J. Autoimmun. 111, 102461 (2020). 32305296 10.1016/j.jaut.2020.102461

[69] E. Stephen-Victor, M. Das, A. Karnam, B. Pitard, J. F. Gautier, J. Bayry, Potential of regulatory T cell-based therapies in the management of severe COVID-19. Eur. Respir. J. 56, 2002182 (2020). 32616599 10.1183/13993003.02182-2020 PMC7331657

[70] C. T. Ellebrecht, V. G. Bhoj, A. Nace, E. J. Choi, X. Mao, M. J. Cho, G. Di Zenzo, A. Lanzavecchia, J. T. Seykora, G. Cotsarelis, M. C. Milone, A. S. Payne, Reengineering chimeric antigen receptor T cells for targeted therapy of autoimmune disease. Science 353, 179–184 (2016). 27365313 10.1126/science.aaf6756 PMC5343513

[71] M. Liao, Y. Liu, J. Yuan, Y. Wen, G. Xu, J. Zhao, L. Cheng, J. Li, X. Wang, F. Wang, L. Liu, I. Amit, S. Zhang, Z. Zhang, Single-cell landscape of bronchoalveolar immune cells in patients with COVID-19. Nat. Med. 26, 842–844 (2020). 32398875 10.1038/s41591-020-0901-9

[72] X. Ren, W. Wen, X. Fan, W. Hou, B. Su, P. Cai, J. Li, Y. Liu, F. Tang, F. Zhang, Y. Yang, J. He, W. Ma, J. He, P. Wang, Q. Cao, F. Chen, Y. Chen, X. Cheng, G. Deng, X. Deng, W. Ding, Y. Feng, R. Gan, C. Guo, W. Guo, S. He, C. Jiang, J. Liang, Y. M. Li, J. Lin, Y. Ling, H. Liu, J. Liu, N. Liu, S. Q. Liu, M. Luo, Q. Ma, Q. Song, W. Sun, G. Wang, F. Wang, Y. Wang, X. Wen, Q. Wu, G. Xu, X. Xie, X. Xiong, X. Xing, H. Xu, C. Yin, D. Yu, K. Yu, J. Yuan, B. Zhang, P. Zhang, T. Zhang, J. Zhao, P. Zhao, J. Zhou, W. Zhou, S. Zhong, X. Zhong, S. Zhang, L. Zhu, P. Zhu, B. Zou, J. Zou, Z. Zuo, F. Bai, X. Huang, P. Zhou, Q. Jiang, Z. Huang, J. X. Bei, L. Wei, X. W. Bian, X. Liu, T. Cheng, X. Li, P. Zhao, F. S. Wang, H. Wang, B. Su, Z. Zhang, K. Qu, X. Wang, J. Chen, R. Jin, Z. Zhang, COVID-19 immune features revealed by a large-scale single-cell transcriptome atlas. Cell 184, 1895–1913.e19 (2021). 33657410 10.1016/j.cell.2021.01.053 PMC7857060

[73] M. Marovich, J. R. Mascola, M. S. Cohen, Monoclonal antibodies for prevention and treatment of COVID-19. JAMA 324, 131–132 (2020). 32539093 10.1001/jama.2020.10245

[74] M. Wang, Q. Yuan, L. Xie, Mesenchymal stem cell-based immunomodulation: Properties and clinical application. Stem Cells Int. 2018, 3057624 (2018). 30013600 10.1155/2018/3057624 PMC6022321

[75] N. Durand, J. Mallea, A. C. Zubair, Insights into the use of mesenchymal stem cells in COVID-19 mediated acute respiratory failure. NPJ Regen. Med. 5, 17 (2020). 33580031 10.1038/s41536-020-00105-z PMC7589470

[76] K. Le Blanc, F. Frassoni, L. Ball, F. Locatelli, H. Roelofs, I. Lewis, E. Lanino, B. Sundberg, M. E. Bernardo, M. Remberger, G. Dini, R. M. Egeler, A. Bacigalupo, W. Fibbe, O. Ringdén, Mesenchymal stem cells for treatment of steroid-resistant, severe, acute graft-versus-host disease: A phase II study. Lancet 371, 1579–1586 (2008). 18468541 10.1016/S0140-6736(08)60690-X

[77] X. Yuan, X. Qin, D. Wang, Z. Zhang, X. Tang, X. Gao, W. Chen, L. Sun, Mesenchymal stem cell therapy induces FLT3L and CD1c^+^ dendritic cells in systemic lupus erythematosus patients. Nat. Commun. 10, 2498 (2019). 31175312 10.1038/s41467-019-10491-8 PMC6555800

[78] S. Sadeghi, S. Soudi, A. Shafiee, S. M. Hashemi, Mesenchymal stem cell therapies for COVID-19: Current status and mechanism of action. Life Sci. 262, 118493 (2020). 32979360 10.1016/j.lfs.2020.118493 PMC7510562

[79] M. A. Canham, J. D. M. Campbell, J. C. Mountford, The use of mesenchymal stromal cells in the treatment of coronavirus disease 2019. J. Transl. Med. 18, 359 (2020). 32958009 10.1186/s12967-020-02532-4 PMC7503434

[80] A. Coelho, R. D. Alvites, M. V. Branquinho, S. G. Guerreiro, A. C. Mauricio, Mesenchymal Stem Cells (MSCs) as a potential therapeutic strategy in COVID-19 patients: Literature research. Front. Cell Dev. Biol. 8, 602647 (2020). 33330498 10.3389/fcell.2020.602647 PMC7710935

[81] M. Kavianpour, M. Saleh, J. Verdi, The role of mesenchymal stromal cells in immune modulation of COVID-19: Focus on cytokine storm. Stem Cell Res. Ther. 11, 404 (2020). 32948252 10.1186/s13287-020-01849-7 PMC7499002

[82] A. Basiri, Z. Pazhouhnia, N. Beheshtizadeh, M. Hoseinpour, A. Saghazadeh, N. Rezaei, Regenerative medicine in COVID-19 treatment: Real opportunities and range of promises. Stem Cell Rev. Rep. 17, 163–175 (2021). 32564256 10.1007/s12015-020-09994-5 PMC7305935

[83] M. S. Choudhery, D. T. Harris, Stem cell therapy for COVID-19: Possibilities and challenges. Cell Biol. Int. 44, 2182–2191 (2020). 32767687 10.1002/cbin.11440 PMC7436138

[84] A. Golchin, Cell-based therapy for severe COVID-19 patients: Clinical trials and cost-utility. Stem Cell Rev. Rep. 17, 56–62 (2021). 33009982 10.1007/s12015-020-10046-1 PMC7532742

[85] A. Golchin, E. Seyedjafari, A. Ardeshirylajimi, Mesenchymal stem cell therapy for COVID-19: Present or future. Stem Cell Rev. Rep. 16, 427–433 (2020). 32281052 10.1007/s12015-020-09973-w PMC7152513

[86] K. Rajarshi, A. Chatterjee, S. Ray, Combating COVID-19 with mesenchymal stem cell therapy. Biotechnol. Rep. (Amst.) 26, e00467 (2020). 32420049 10.1016/j.btre.2020.e00467 PMC7224671

[87] R. N. Riedel, A. Perez-Perez, V. Sanchez-Margalet, C. L. Varone, J. L. Maymo, Stem cells and COVID-19: Are the human amniotic cells a new hope for therapies against the SARS-CoV-2 virus? Stem Cell Res. Ther. 12, 155 (2021). 33648582 10.1186/s13287-021-02216-w PMC7919997

[88] S. H. Wang, A. K. Shetty, K. Jin, R. Chunhua Zhao, Combating COVID-19 with mesenchymal stem/stromal cell therapy: Promise and challenges. Front. Cell Dev. Biol. 8, 627414 (2020). 33469541 10.3389/fcell.2020.627414 PMC7813676

[89] B. L. Yen, M. L. Yen, L. T. Wang, K. J. Liu, H. K. Sytwu, Current status of mesenchymal stem cell therapy for immune/inflammatory lung disorders: Gleaning insights for possible use in COVID-19. Stem Cells Transl. Med. 9, 1163–1173 (2020). 32526079 10.1002/sctm.20-0186 PMC7300965

[90] C. M. Romero-Sanchez, I. Diaz-Maroto, E. Fernandez-Diaz, A. Sanchez-Larsen, A. Layos-Romero, J. Garcia-Garcia, E. Gonzalez, I. Redondo-Penas, A. B. Perona-Moratalla, J. A. Del Valle-Perez, J. Gracia-Gil, L. Rojas-Bartolome, I. Feria-Vilar, M. Monteagudo, M. Palao, E. Palazon-Garcia, C. Alcahut-Rodriguez, D. Sopelana-Garay, Y. Moreno, J. Ahmad, T. Segura, Neurologic manifestations in hospitalized patients with COVID-19: The ALBACOVID registry. Neurology 95, e1060–e1070 (2020). 32482845 10.1212/WNL.0000000000009937 PMC7668545

[91] M. A. Ellul, L. Benjamin, B. Singh, S. Lant, B. D. Michael, A. Easton, R. Kneen, S. Defres, J. Sejvar, T. Solomon, Neurological associations of COVID-19. Lancet Neurol. 19, 767–783 (2020). 32622375 10.1016/S1474-4422(20)30221-0 PMC7332267

[92] G. Das, N. Mukherjee, S. Ghosh, Neurological insights of COVID-19 pandemic. ACS Chem. Nerosci. 11, 1206–1209 (2020). 10.1021/acschemneuro.0c00201 32320211

[93] R. Chen, K. Wang, J. Yu, Z. Chen, C. Wen, Z. Xu, The spatial and cell-type distribution of SARS-CoV-2 receptor ACE2 in the human and mouse brains. Front. Neurol. 11, 573095 (2020). 33551947 10.3389/fneur.2020.573095 PMC7855591

[94] K. Bohmwald, N. M. S. Galvez, M. Rios, A. M. Kalergis, Neurologic alterations due to respiratory virus infections. Front. Cell. Neurosci. 12, 386 (2018). 30416428 10.3389/fncel.2018.00386 PMC6212673

[95] D. H. Brann, T. Tsukahara, C. Weinreb, M. Lipovsek, K. Van den Berge, B. Gong, R. Chance, I. C. Macaulay, H.-J. Chou, R. B. Fletcher, D. Das, K. Street, H. R. de Bezieux, Y.-G. Choi, D. Risso, S. Dudoit, E. Purdom, J. Mill, R. A. Hachem, H. Matsunami, D. W. Logan, B. J. Goldstein, M. S. Grubb, J. Ngai, S. R. Datta, Non-neuronal expression of SARS-CoV-2 entry genes in the olfactory system suggests mechanisms underlying COVID-19-associated anosmia. Sci. Adv. 6, eabc5801 (2020). 32937591 10.1126/sciadv.abc5801 PMC10715684

[96] R. Beyrouti, M. E. Adams, L. Benjamin, H. Cohen, S. F. Farmer, Y. Y. Goh, F. Humphries, H. R. Jager, N. A. Losseff, R. J. Perry, S. Shah, R. J. Simister, D. Turner, A. Chandratheva, D. J. Werring, Characteristics of ischaemic stroke associated with COVID-19. J. Neurol. Neurosurg. Psychiatry 91, 889–891 (2020). 32354768 10.1136/jnnp-2020-323586 PMC7231545

[97] A. Sweid, B. Hammoud, J. H. Weinberg, M. Oneissi, E. Raz, M. Shapiro, M. DePrince, S. Tjoumakaris, M. R. Gooch, N. A. Herial, H. Zarzour, V. Romo, R. H. Rosenwasser, P. Jabbour, Letter: Thrombotic neurovascular disease in COVID-19 patients. Neurosurgery 87, E400–E406 (2020). 32496534 10.1093/neuros/nyaa254 PMC7313768

[98] L. Zanin, G. Saraceno, P. P. Panciani, G. Renisi, L. Signorini, K. Migliorati, M. M. Fontanella, SARS-CoV-2 can induce brain and spine demyelinating lesions. Acta Neurochir. 162, 1491–1494 (2020). 32367205 10.1007/s00701-020-04374-x PMC7197630

[99] A. Thiruvalluvan, M. Czepiel, Y. A. Kap, I. Mantingh-Otter, I. Vainchtein, J. Kuipers, M. Bijlard, W. Baron, B. Giepmans, W. Brück, B. A. ‘t Hart, E. Boddeke, S. Copray, Survival and functionality of human induced pluripotent stem cell-derived oligodendrocytes in a nonhuman primate model for multiple sclerosis. Stem Cells Transl. Med. 5, 1550–1561 (2016). 27400790 10.5966/sctm.2016-0024 PMC5070510

[100] M. Krause, T. G. Phan, H. Ma, C. G. Sobey, R. Lim, Cell-based therapies for stroke: Are we there yet? Front. Neurol. 10, 656 (2019). 31293500 10.3389/fneur.2019.00656 PMC6603096

[101] Y. Kang, T. Chen, D. Mui, V. Ferrari, D. Jagasia, M. Scherrer-Crosbie, Y. Chen, Y. Han, Cardiovascular manifestations and treatment considerations in covid-19. Heart 106, 1132–1141 (2020). 32354800 10.1136/heartjnl-2020-317056 PMC7211105

[102] D. Wang, B. Hu, C. Hu, F. Zhu, X. Liu, J. Zhang, B. Wang, H. Xiang, Z. Cheng, Y. Xiong, Y. Zhao, Y. Li, X. Wang, Z. Peng, Clinical characteristics of 138 hospitalized patients with 2019 novel coronavirus-infected pneumonia in Wuhan, China. JAMA 323, 1061–1069 (2020). 32031570 10.1001/jama.2020.1585 PMC7042881

[103] J. A. Perez-Bermejo, S. Kang, S. J. Rockwood, C. R. Simoneau, D. A. Joy, A. C. Silva, G. N. Ramadoss, W. R. Flanigan, P. Fozouni, H. Li, P. Y. Chen, K. Nakamura, J. D. Whitman, P. J. Hanson, B. M. McManus, M. Ott, B. R. Conklin, T. C. McDevitt, SARS-CoV-2 infection of human iPSC-derived cardiac cells reflects cytopathic features in hearts of patients with COVID-19. Sci. Transl. Med. 13, eabf7872 (2021). 33723017 10.1126/scitranslmed.abf7872 PMC8128284

[104] Y. J. Geng, Z. Y. Wei, H. Y. Qian, J. Huang, R. Lodato, R. J. Castriotta, Pathophysiological characteristics and therapeutic approaches for pulmonary injury and cardiovascular complications of coronavirus disease 2019. Cardiovasc. Pathol. 47, 107228 (2020). 32375085 10.1016/j.carpath.2020.107228 PMC7162778

[105] G. P. Meyer, K. C. Wollert, J. Lotz, J. Steffens, P. Lippolt, S. Fichtner, H. Hecker, A. Schaefer, L. Arseniev, B. Hertenstein, A. Ganser, H. Drexler, Intracoronary bone marrow cell transfer after myocardial infarction: Eighteen months’ follow-up data from the randomized, controlled BOOST (BOne marrOw transfer to enhance ST-elevation infarct regeneration) trial. Circulation 113, 1287–1294 (2006). 16520413 10.1161/CIRCULATIONAHA.105.575118

[106] D. M. Leistner, U. Fischer-Rasokat, J. Honold, F. H. Seeger, V. Schachinger, R. Lehmann, H. Martin, I. Burck, C. Urbich, S. Dimmeler, A. M. Zeiher, B. Assmus, Transplantation of progenitor cells and regeneration enhancement in acute myocardial infarction (TOPCARE-AMI): Final 5-year results suggest long-term safety and efficacy. Clin. Res. Cardiol. 100, 925–934 (2011). 21633921 10.1007/s00392-011-0327-y

[107] K. C. Wollert, G. P. Meyer, J. Muller-Ehmsen, C. Tschope, V. Bonarjee, A. I. Larsen, A. E. May, K. Empen, E. Chorianopoulos, U. Tebbe, J. Waltenberger, H. Mahrholdt, B. Ritter, J. Pirr, D. Fischer, M. Korf-Klingebiel, L. Arseniev, H. G. Heuft, J. E. Brinchmann, D. Messinger, B. Hertenstein, A. Ganser, H. A. Katus, S. B. Felix, M. P. Gawaz, K. Dickstein, H. P. Schultheiss, D. Ladage, S. Greulich, J. Bauersachs, Intracoronary autologous bone marrow cell transfer after myocardial infarction: The BOOST-2 randomised placebo-controlled clinical trial. Eur. Heart J. 38, 2936–2943 (2017). 28431003 10.1093/eurheartj/ehx188

[108] J. H. Traverse, T. D. Henry, C. J. Pepine, J. T. Willerson, D. X. Zhao, S. G. Ellis, J. R. Forder, R. D. Anderson, A. K. Hatzopoulos, M. S. Penn, E. C. Perin, J. Chambers, K. W. Baran, G. Raveendran, C. Lambert, A. Lerman, D. I. Simon, D. E. Vaughan, D. Lai, A. P. Gee, D. A. Taylor, C. R. Cogle, J. D. Thomas, R. E. Olson, S. Bowman, J. Francescon, C. Geither, E. Handberg, C. Kappenman, L. Westbrook, L. B. Piller, L. M. Simpson, S. Baraniuk, C. Loghin, D. Aguilar, S. Richman, C. Zierold, D. B. Spoon, J. Bettencourt, S. L. Sayre, R. W. Vojvodic, S. I. Skarlatos, D. J. Gordon, R. F. Ebert, M. Kwak, L. A. Moye, R. D. Simari; Cardiovascular Cell Therapy Research Network (CCTRN), Effect of the use and timing of bone marrow mononuclear cell delivery on left ventricular function after acute myocardial infarction: The TIME randomized trial. JAMA 308, 2380–2389 (2012). 23129008 10.1001/jama.2012.28726 PMC3652242

[109] R. Bolli, J. M. Hare, K. L. March, C. J. Pepine, J. T. Willerson, E. C. Perin, P. C. Yang, T. D. Henry, J. H. Traverse, R. D. Mitrani, A. Khan, I. Hernandez-Schulman, D. A. Taylor, D. L. DiFede, J. A. C. Lima, A. Chugh, J. Loughran, R. W. Vojvodic, S. L. Sayre, J. Bettencourt, M. Cohen, L. Moye, R. F. Ebert, R. D. Simari; Cardiovascular Cell Therapy Research Network (CCTRN), Rationale and design of the CONCERT-HF trial (Combination of Mesenchymal and c-kit^+^ Cardiac Stem Cells As Regenerative Therapy for Heart Failure). Circ. Res. 122, 1703–1715 (2018). 29703749 10.1161/CIRCRESAHA.118.312978 PMC5993622

[110] P. Menasché, V. Vanneaux, A. Hagege, A. Bel, B. Cholley, A. Parouchev, I. Cacciapuoti, R. Al-Daccak, N. Benhamouda, H. Blons, O. Agbulut, L. Tosca, J. H. Trouvin, J. R. Fabreguettes, V. Bellamy, D. Charron, E. Tartour, G. Tachdjian, M. Desnos, J. Larghero, Transplantation of human embryonic stem cell-derived cardiovascular progenitors for severe ischemic left ventricular dysfunction. J. Am. Coll. Cardiol. 71, 429–438 (2018). 29389360 10.1016/j.jacc.2017.11.047

[111] D. Cyranoski, ‘Reprogrammed’ stem cells approved to mend human hearts for the first time. Nature 557, 619–620 (2018). 29844563 10.1038/d41586-018-05278-8

[112] S. Singh, T. Chakravarty, P. Chen, A. Akhmerov, J. Falk, O. Friedman, T. Zaman, J. E. Ebinger, M. Gheorghiu, L. Marban, E. Marban, R. R. Makkar, Allogeneic cardiosphere-derived cells (CAP-1002) in critically ill COVID-19 patients: Compassionate-use case series. Basic Res. Cardiol. 115, 36 (2020). 32399655 10.1007/s00395-020-0795-1 PMC7214858

[113] S. C. Ng, H. Tilg, COVID-19 and the gastrointestinal tract: More than meets the eye. Gut 69, 973–974 (2020). 32273292 10.1136/gutjnl-2020-321195 PMC7211058

[114] N. Chen, M. Zhou, X. Dong, J. Qu, F. Gong, Y. Han, Y. Qiu, J. Wang, Y. Liu, Y. Wei, J. Xia, T. Yu, X. Zhang, L. Zhang, Epidemiological and clinical characteristics of 99 cases of 2019 novel coronavirus pneumonia in Wuhan, China: A descriptive study. Lancet 395, 507–513 (2020). 32007143 10.1016/S0140-6736(20)30211-7 PMC7135076

[115] C. Zhang, L. Shi, F. S. Wang, Liver injury in COVID-19: Management and challenges. Lancet Gastroenterol. Hepatol. 5, 428–430 (2020). 32145190 10.1016/S2468-1253(20)30057-1 PMC7129165

[116] X. Chai, L. Hu, Y. Zhang, W. Han, Z. Lu, A. Ke, J. Zhou, G. Shi, N. Fang, J. Fan, J. Cai, J. Fan, F. Lan, Specific ACE2 expression in cholangiocytes may cause liver damage after 2019-nCoV infection. bioRxiv 2020.2002.2003.931766. [**Preprint**]. 4 February 2020. 10.1101/2020.02.03.931766.

[117] S. H. Wong, R. N. Lui, J. J. Sung, Covid-19 and the digestive system. J. Gastroenterol. Hepatol. 35, 744–748 (2020). 32215956 10.1111/jgh.15047

[118] G. Feng, K. I. Zheng, Q.-Q. Yan, R. S. Rios, G. Targher, C. D. Byrne, S. V. Poucke, W.-Y. Liu, M.-H. Zheng, COVID-19 and liver dysfunction: Current insights and emergent therapeutic strategies. J. Clin. Transl. Hepatol. 8, 18–24 (2020). 32274342 10.14218/JCTH.2020.00018 PMC7132016

[119] R. C. Huebert, J. Rakela, Cellular therapy for liver disease. Mayo Clin. Proc. 89, 414–424 (2014). 24582199 10.1016/j.mayocp.2013.10.023 PMC4212517

[120] S. C. Strom, R. A. Fisher, M. T. Thompson, A. J. Sanyal, P. E. Cole, J. M. Ham, M. P. Posner, Hepatocyte transplantation as a bridge to orthotopic liver transplantation in terminal liver failure. Transplantation 63, 559–569 (1997). 9047152 10.1097/00007890-199702270-00014

[121] C. Ronco, T. Reis, F. Husain-Syed, Management of acute kidney injury in patients with COVID-19. Lancet Respir. Med. 8, 738–742 (2020). 32416769 10.1016/S2213-2600(20)30229-0 PMC7255232

[122] G. Pei, Z. Zhang, J. Peng, L. Liu, C. Zhang, C. Yu, Z. Ma, Y. Huang, W. Liu, Y. Yao, R. Zeng, G. Xu, Renal involvement and early prognosis in patients with COVID-19 pneumonia. J. Am. Soc. Nephrol. 31, 1157–1165 (2020). 32345702 10.1681/ASN.2020030276 PMC7269350

[123] B. Diao, C. Wang, R. Wang, Z. Feng, J. Zhang, H. Yang, Y. Tan, H. Wang, C. Wang, L. Liu, Y. Liu, Y. Liu, G. Wang, Z. Yuan, X. Hou, L. Ren, Y. Wu, Y. Chen, Human kidney is a target for novel severe acute respiratory syndrome coronavirus 2 infection. 12, 2506 (2020). 10.1038/s41467-021-22781-1 PMC8096808 33947851

[124] C. Rota, M. Morigi, B. Imberti, Stem cell therapies in kidney diseases: Progress and challenges. Int. J. Mol. Sci. 20, 2790 (2019). 10.3390/ijms20112790 PMC6600599 31181604

[125] M. Swaminathan, M. Stafford-Smith, G. M. Chertow, D. G. Warnock, V. Paragamian, R. M. Brenner, F. Lellouche, A. Fox-Robichaud, M. G. Atta, S. Melby, R. L. Mehta, R. Wald, S. Verma, C. D. Mazer; ACT-AKI investigators, Allogeneic mesenchymal stem cells for treatment of AKI after cardiac surgery. J. Am. Soc. Nephrol. 29, 260–267 (2018). 29038286 10.1681/ASN.2016101150 PMC5748899

[126] E. Terpos, I. Ntanasis-Stathopoulos, I. Elalamy, E. Kastritis, T. N. Sergentanis, M. Politou, T. Psaltopoulou, G. Gerotziafas, M. A. Dimopoulos, Hematological findings and complications of COVID-19. Am. J. Hematol. 95, 834–847 (2020). 32282949 10.1002/ajh.25829 PMC7262337

[127] T. J. Oxley, J. Mocco, S. Majidi, C. P. Kellner, H. Shoirah, I. P. Singh, R. A. De Leacy, T. Shigematsu, T. R. Ladner, K. A. Yaeger, M. Skliut, J. Weinberger, N. S. Dangayach, J. B. Bederson, S. Tuhrim, J. T. Fifi, Large-vessel stroke as a presenting feature of Covid-19 in the young. N. Engl. J. Med. 382, e60 (2020). 32343504 10.1056/NEJMc2009787 PMC7207073

[128] F. A. Klok, M. J. H. A. Kruip, N. J. M. van der Meer, M. S. Arbous, D. A. M. P. J. Gommers, K. M. Kant, F. H. J. Kaptein, J. van Paassen, M. A. M. Stals, M. V. Huisman, H. Endeman, Incidence of thrombotic complications in critically ill ICU patients with COVID-19. Thromb. Res. 191, 145–147 (2020). 32291094 10.1016/j.thromres.2020.04.013 PMC7146714

[129] N. Tang, D. Li, X. Wang, Z. Sun, Abnormal coagulation parameters are associated with poor prognosis in patients with novel coronavirus pneumonia. J. Thromb. Haemost. 18, 844–847 (2020). 32073213 10.1111/jth.14768 PMC7166509

[130] J. M. Connors, J. H. Levy, COVID-19 and its implications for thrombosis and anticoagulation. Blood 135, 2033–2040 (2020). 32339221 10.1182/blood.2020006000 PMC7273827

[131] Z. Varga, A. J. Flammer, P. Steiger, M. Haberecker, R. Andermatt, A. S. Zinkernagel, M. R. Mehra, R. A. Schuepbach, F. Ruschitzka, H. Moch, Endothelial cell infection and endotheliitis in COVID-19. Lancet 395, 1417–1418 (2020). 32325026 10.1016/S0140-6736(20)30937-5 PMC7172722

[132] W. K. Sietsema, A. Kawamoto, H. Takagi, D. W. Losordo, Autologous CD34+ cell therapy for ischemic tissue repair. Circ. J. 83, 1422–1430 (2019). 31178469 10.1253/circj.CJ-19-0240

[133] S. H. Abd-Allah, S. M. Shalaby, E. Abd-Elbary, A. A. Saleh, M. A. El-Magd, Human peripheral blood CD34+ cells attenuate oleic acid-induced acute lung injury in rats. Cytotherapy 17, 443–453 (2015). 25536864 10.1016/j.jcyt.2014.11.002

[134] X. Huang, K. Sun, Y. D. Zhao, S. M. Vogel, Y. Song, N. Mahmud, Y. Y. Zhao, Human CD34+ progenitor cells freshly isolated from umbilical cord blood attenuate inflammatory lung injury following LPS challenge. PLOS ONE 9, e88814 (2014). 24558433 10.1371/journal.pone.0088814 PMC3928308

[135] M. Hassanpour, J. Rezaie, M. Nouri, Y. Panahi, The role of extracellular vesicles in COVID-19 virus infection. Infect. Genet. Evol. 85, 104422 (2020). 32544615 10.1016/j.meegid.2020.104422 PMC7293471

[136] L. Rezakhani, A. F. Kelishadrokhi, A. Soleimanizadeh, S. Rahmati, Mesenchymal stem cell (MSC)-derived exosomes as a cell-free therapy for patients Infected with COVID-19: Real opportunities and range of promises. Chem. Phys. Lipids 234, 105009 (2021). 33189639 10.1016/j.chemphyslip.2020.105009 PMC7658620

[137] G. Pironti, D. C. Andersson, L. H. Lund, Mechanistic and therapeutic implications of extracellular vesicles as a potential link between Covid-19 and cardiovascular disease manifestations. Front. Cell Dev. Biol. 9, 640723 (2021). 33644077 10.3389/fcell.2021.640723 PMC7905102

[138] G. Pocsfalvi, R. Mammadova, A. P. Ramos Juarez, R. Bokka, F. Trepiccione, G. Capasso, COVID-19 and extracellular vesicles: An intriguing interplay. Kidney Blood Press. Res. 45, 661–670 (2020). 32957112 10.1159/000511402 PMC7573892

[139] L. L. Popplewell, S. J. Forman, Is there an upper age limit for bone marrow transplantation? Bone Marrow Transplant. 29, 277–284 (2002). 11896423 10.1038/sj.bmt.1703382

[140] S. Golpanian, J. El-Khorazaty, A. Mendizabal, D. L. DiFede, V. Y. Suncion, V. Karantalis, J. E. Fishman, E. Ghersin, W. Balkan, J. M. Hare, Effect of aging on human mesenchymal stem cell therapy in ischemic cardiomyopathy patients. J. Am. Coll. Cardiol. 65, 125–132 (2015). 25593053 10.1016/j.jacc.2014.10.040 PMC4405121

[141] H. Brave, R. MacLoughlin, State of the art review of cell therapy in the treatment of lung disease, and the potential for aerosol delivery. Int. J. Mol. Sci. 21, 6435 (2020). 10.3390/ijms21176435 PMC7503246 32899381

[142] S. Mahendiratta, S. Bansal, P. Sarma, H. Kumar, G. Choudhary, S. Kumar, A. Prakash, R. Sehgal, B. Medhi, Stem cell therapy in COVID-19: Pooled evidence from SARS-CoV-2, SARS-CoV, MERS-CoV and ARDS: A systematic review. Biomed. Pharmacother. 137, 111300 (2021). 33529945 10.1016/j.biopha.2021.111300 PMC7843034

[143] S. Pati, M. H. Gerber, T. D. Menge, K. A. Wataha, Y. Zhao, J. A. Baumgartner, J. Zhao, P. A. Letourneau, M. P. Huby, L. A. Baer, J. R. Salsbury, R. A. Kozar, C. E. Wade, P. A. Walker, P. K. Dash, C. S. Cox Jr., M. F. Doursout, J. B. Holcomb, Bone marrow derived mesenchymal stem cells inhibit inflammation and preserve vascular endothelial integrity in the lungs after hemorrhagic shock. PLOS ONE 6, e25171 (2011). 21980392 10.1371/journal.pone.0025171 PMC3182198

[144] C. Premer, A. Blum, M. A. Bellio, I. H. Schulman, B. E. Hurwitz, M. Parker, C. R. Dermarkarian, D. L. DiFede, W. Balkan, A. Khan, J. M. Hare, Allogeneic mesenchymal stem cells restore endothelial function in heart failure by stimulating endothelial progenitor cells. EBioMedicine 2, 467–475 (2015). 26137590 10.1016/j.ebiom.2015.03.020 PMC4485912

[145] J. D. Boeke, G. Church, A. Hessel, N. J. Kelley, A. Arkin, Y. Cai, R. Carlson, A. Chakravarti, V. W. Cornish, L. Holt, F. J. Isaacs, T. Kuiken, M. Lajoie, T. Lessor, J. Lunshof, M. T. Maurano, L. A. Mitchell, J. Rine, S. Rosser, N. E. Sanjana, P. A. Silver, D. Valle, H. Wang, J. C. Way, L. Yang, The Genome Project-write. Science 353, 126–127 (2016). 27256881 10.1126/science.aaf6850

[146] A. H. M. Ng, P. Khoshakhlagh, J. E. Rojo Arias, G. Pasquini, K. Wang, A. Swiersy, S. L. Shipman, E. Appleton, K. Kiaee, R. E. Kohman, A. Vernet, M. Dysart, K. Leeper, W. Saylor, J. Y. Huang, A. Graveline, J. Taipale, D. E. Hill, M. Vidal, J. M. Melero-Martin, V. Busskamp, G. M. Church, A comprehensive library of human transcription factors for cell fate engineering. Nat. Biotechnol. 39, 510–519 (2021). 33257861 10.1038/s41587-020-0742-6 PMC7610615

